# Genome-wide comprehensive analysis the molecular phylogenetic evolution, functional divergence and tissue-specific expression of *GH3* gene family in *Salvia miltiorrhiza, Arabidopsis thaliana*, and Oryza sativa

**DOI:** 10.3389/fpls.2025.1644853

**Published:** 2025-11-14

**Authors:** Bin Wang, Min Li, Jun Chen, Longtao Chen

**Affiliations:** 1 College of Life Sciences, Henan Normal University, Xinxiang, China; 2 School of Science, East China University of Technology, Nanchang, China; 3 Life Science & Technology School, Lingnan Normal University, Zhanjiang, China

**Keywords:** conserved motifs, functional divergence, GH3 gene family, nonsynonymous and synonymous substitution rate, phylogenetic analysis, positive selection, tissue-specific expression

## Abstract

Auxin, as a central phytohormone and signaling molecule, plays a crucial role in plant growth and development. The activity of auxin is tightly regulated by the auxin-responsive *GH3* gene family. In this study, a total of 40 *GH3* genes in *A. thaliana*, *S. miltiorrhiza*, and *O. sativa* were identified and subjected to comprehensive study. Phylogenetic analysis revealed that those *GH3* genes can be classified into three distinct subgroups, with 11 pairs of paralogs identified. Genetic divergence analysis indicated that the *GH3* gene family had predominantly experienced purifying selection as evidenced by the Ka/Ks ratio being less than 1 for all 11 paralogs pairs. Positive selection analysis with the site and branch-site models further suggested that *SmGH3 AtGH3* and *OsGH3* genes had gone through purifying selective pressure for adaptive evolution. Motif analysis indicated that group-specific motifs may contribute to functional divergence across species and subgroups. Functional divergence analysis confirmed that subgroup-specific genes have experienced functional divergence during evolution, and elucidated the molecular mechanisms underlying their divergent functions. The tissue-specific expression analysis of *SmGH3 AtGH3* and *OsGH3* genes revealed that these genes might perform distinct functions in different tissues. This study performed a comprehensive bioinformatics analysis of the *GH3* gene family, offering valuable information to further elucidate the functional roles of *GH3* genes.

## 1 Introduction

Auxin plays a crucial role in regulating various aspects of plant development and growth, such as apical dominance, auxin transport, shoot elongation, and plant metabolism (Woodward and Bartel, 2005; Baranwal et al., 2017). A wide array of these processes are modulated by auxin-responsive genes, primarily including Auxin Response Factor (ARF) genes, which function as transcriptional activators, Small Auxin Up RNA (SAUR) genes, which regulate auxin signaling pathways, Auxin/Indole-3-Acetic Acid (AUX/IAA) genes, which act as transcriptional repressors, and Gretchen Hagen 3 (GH3) genes, which regulate the dynamic process of endogenous auxin homeostasis (Hagen and Guilfoyle, 1984; Fu et al., 2011; Chen et al., 2014; Kong et al., 2019). Moreover, GH3-mediated auxin regulation constitutes an essential component of the intricate network of auxin activity that governs plant responses to environmental stresses (Wang et al., 2010). Thus, the *GH3* gene family primarily comprises a series of genes that encode specific enzymes, which capable of conjugating various amino acids to chemically diverse compounds (Kong et al., 2019). Typically, the *GH3* gene family is significantly influenced by various hormones, including growth-promoting hormones such as brassinosteroids (BRs) and gibberellins (GA), stress-related hormones like abscisic acid (ABA), jasmonic acid (JA), and salicylic acid (SA), as well as the ripening/senescence-associated hormone Ethylene (ETH). Besides, these genes are responsive to biotic stresses caused by pathogens and abiotic stress factors such as light, salt, drought, cold, and other environmental stresses (Shin-Ichiro et al., 2002; Park et al., 2007; Zhang et al., 2009; Takase et al., 2010; Wang et al., 2010).

The first *GH3* gene was identified in soybean (*Glycine max*) through differential hybridization analysis, where it was found to be responsive to the plant hormone auxin (Hagen and Guilfoyle, 1984). Since then, *GH3* genes have been reported in various plant species (Terol et al., 2006; Okrent and Wildermuth, 2011). The *GH3* gene family was first characterized in the model organism *Arabidopsis thaliana (A. thaliana)*, with 19 distinct members identified (Staswick et al., 2002). Through genome-wide analysis, an increasing number of *GH3* genes family have been successively identified in diverse species, including 13 in *Oryza sativa* (*O. sativa*) (Jain et al., 2006a), 9 in grape (Bottcher et al., 2011), 13 in maize (Feng et al., 2015), 15 in rosids (Okrent and Wildermuth, 2011), 15 in tomato (Kumar et al., 2012), and in legumes (11 in chickpea, 28 in soybean, 10 in *Medicago*, and 18 in *Lotus*) (Singh et al., 2014), 15 in apple (Yuan et al., 2013), and 10 in Melon (Chen et al., 2023). The number of *GH3* genes varies across different plant species and is closely associated with the expansion and diversification of the gene family. However, the evolutionary dynamics and molecular mechanisms underlying the diversification of the *GH3* gene family remain poorly understood. Elucidating these mechanisms is essential for understanding the roles of *GH3* genes in plant evolution and adaptation.

Based on the sequence similarity and substrate specificity analyses, GH3 proteins are typically classified into three distinct clades (I, II, and III) using distance-based phylogenetic methods (Staswick et al., 2002, 2005; Okrent et al., 2009). Proteins in clade I primarily exhibit JA- or SA-amido synthetase activity, using JA or SA as substrates to synthesize corresponding amide conjugates such as JA-Ile or SA-amide compounds (Staswick et al., 2002). Proteins from clade II are responsive to auxin and exhibit auxin-inducible expression profiles (Staswick et al., 2005). The functions of most clade III GH3 proteins remain not yet fully characterized (Kong et al., 2019), although certain clade III proteins have been found to be induced in response to infection by *Pseudomonas syringae* (Nobuta et al., 2007; Okrent et al., 2009). The functional divergence observed during the phylogenetic evolution of *GH3* genes is notable, and this functional specialization undoubtedly underscores the complexity of the molecular mechanism involved. However, the molecular mechanisms underlying these functional divergences remain unexplored and require further investigation.

*S. miltiorrhiza* a well-known member of traditional Chinese medicine, is widely utilized for treating various cerebrovascular and cardiovascular disorders (Zhou et al., 2005; Geng et al., 2015). The pharmacological activities of *S. miltiorrhiza* can be attributed to its two primary bioactive components. One group consists of lipid-soluble diterpenoids, commonly referred to as tanshinones, while the other comprises water-soluble phenolic acids, such as rosmarinic acid (RA) and salvianolic acid B (Sal B) (Ma et al., 2012; Wang et al., 2018). The biosynthetic pathways responsible for the production of these two pharmacologically active components in *S. miltiorrhiza* are modulated by various plant hormone signals, including SA, JA, IAA and ABA, which collectively influence the synthesis and accumulation of its medicinal constituents (Zhao et al., 2010; Cui et al., 2012; Ge et al., 2015; Huang et al., 2023). Additionally, members of the GH3 protein family play a crucial role in maintaining auxin homeostasis through enzymatic catalysis of amino acid conjugation to phytohormones such as IAA, SA, and JA (Staswick et al., 2002, 2005). These enzymatic reaction processes and their products may be associated with the regulation of hormone signaling pathways and secondary metabolism in *S. miltiorrhiza*. Due to its relatively small genome and well-characterized secondary metabolic pathways, *S. miltiorrhiza* is recommended by many researchers as a model medicinal plant in the field of medicinal plant research (Ma et al., 2012; Xu et al., 2015). Likewise, *A. thaliana* is widely recognized as a model organism in plant research due to its small genome size and short growth cycle. Similarly, *O. sativa* has been established as a genetic, molecular, and functional model organism for research due to its importance as a major food crop (Yang et al., 2008). The objective of our study is to perform a comprehensive phylogenomic analysis of the *GH3* gene family across three plant species, with the aim of elucidating the molecular evolution mechanisms underlying their evolutionary dynamics, genetic and functional divergence of this gene family during their evolution.

Recent advancements in high-throughput sequencing technologies have significantly expanded access to plant genome and transcriptome databases, providing robust analytical platforms for studying gene expression and functional characterization. Expressed sequence tags (ESTs), short cDNA fragments derived from various tissues, represent partial sequences of transcribed genes and provide valuable tools for analyzing mRNA expression profiles (Ohlrogge and Benning, 2000). Thus, the relative frequency of ESTs or full-length cDNAs across different databases serves as an efficient tool for preliminary analysis of gene expression patterns in different tissues (Adams et al., 1995). Therefore, the transcriptomes of different tissues from *S. miltiorrhiza* (Zhang et al., 2015) and the GenBank EST databases (https://www.ncbi.nlm.nih.gov/genbank/dbest/) (Boguski et al., 1993) for *A. thaliana* and *O. sativa* collectively provide comprehensive insights into genetic transcription, thereby enabling more systematic and detailed analyses of *GH3* gene expression across these species.

In this study, following the identification of the *GH3* gene family in *S. miltiorrhiza*, we conducted a comprehensive bioinformatics analysis of the *GH3* gene families across *A. thaliana*, *S. miltiorrhiza*, and *O. sativa*. We first examined the conserved domains, gene structure, motifs and *cis*-regulatory elements of those genes. Subsequently, we constructed a phylogenetic tree to evaluate the evolutionary relationships among the *SmGH3*, *AtGH3*, and *OsGH3* genes. To investigate the selective pressures driving gene divergence, we calculated the Ka/Ks ratios for paralogous gene pairs. Using site and branch-site models, we detected positive selection in the *SmGH3*, *AtGH3*, and *OsGH3* genes with the PAML program. Additionally, we analyzed functional divergence among the *SmGH3*, *AtGH3*, and *OsGH3* genes using the DIVERGE program. Finally, the tissue-specific expression patterns of the *SmGH3*, *AtGH3*, and *OsGH3* genes were preliminarily evaluated using the EST database and RNA-seq data.

## 2 Methods

### 2.1 Identification of the members of *GH3* in *S. miltiorrhiza*, *A. thaliana*, and *O. sativa*

Using the TAIR (The Arabidopsis Information Resource) database (Garcia-Hernandez et al., 2002) and RAP (Rice Annotation Project) database (Sakai et al., 2013), we obtained the amino acids sequences of 19 *AtGH3* and 13 *OsGH3*, respectively. Then, employing the BioEdit software (Hall, 1999), we used these amino acid sequences as queries to search the current *S. miltiorrhiza* genome assembly, which covers approximately 92% of the entire genome and 96% of the protein-coding genes (Song et al., 2013; Xu et al., 2016) under the BLASTp program (Altschul et al., 1997). Finally, following the previously established methods for screening gene family members (Wang et al., 2017, 2019) and the basic characteristics of GH3 protein, we identified the *GH3* gene family in *S. miltiorrhiza*. The theoretical isoelectric point (*pI*) and molecular weight (Mw) of the SmGH3, AtGH3, and OsGH3 proteins were determined using the Compute pI/Mw tool on the ExPASy server (Wilkins et al., 1999).

### 2.2 Multiple sequence alignment and phylogenetic analyses

Using the Gblocks_0.91b program (Castresana, 2000), we initially identified the conserved block of SmGH3, AtGH3, and OsGH3 proteins. Subsequently, a multiple sequence alignment of the conserved SmGH3, AtGH3, and OsGH3 proteins was performed using the DNAMAN program (Lynnon Corporation, San Ramon, CA, USA). Additionally, to determine sequence identities, pairwise comparisons were conducted using the MegAlign package of the DNAStar program with the SmGH3, AtGH3, and OsGH3 amino acid sequences. Furthermore, to investigate the evolutionary relationships among *SmGH3*, *AtGH3*, and *OsGH3* genes, as well as to identify the orthologs and paralogs among these genes in thses three species, an unrooted tree was generated using Bayesian inference implemented in MrBayes (Huelsenbeck and Ronquist, 2001; Hall, 2005) based on the SmGH3, AtGH3, and OsGH3 amino acid sequences. The substitution model employed for the construction of the phylogenetic tree was JTT + I + G, which was selected using the PhyloSuite v1.2.1 program (Zhang et al., 2019). The phylogenetic tree was represented with the help of Treeview1.61 software (Zhai et al., 2002).

### 2.3 Gene structure analysis and motif detection

The gene structure of *SmGH3*, *AtGH3*, and *OsGH3* was analyzed using the Gene Structure Display Server software (Guo et al., 2007), based on their respective coding sequences and corresponding gene sequences. The conserved motifs were identified in SmGH3, AtGH3, and OsGH3 proteins using the online MEME tool with the following parameters: the e-values less than 2 x 10^-30^, and any number of repetitions of a motif (Bailey et al., 2006; Zhang et al., 2015).

### 2.4 *Cis*-regulatory elements in the promoter regions analysis

To investigate the regulatory information of gene expression, we conducted a comprehensive analysis of *cis*-regulatory elements across the promoter regions of the *SmGH3*, *AtGH3*, and *OsGH3* genes. We extracted the 2.0 kb upstream sequence of the start codon (ATG) for each of these genes from their respective genomic scaffolds. Subsequently, we utilized the online platform of PlantCARE database (Lescot et al., 2002) to predict the *cis*-regulatory elements present in these promoter regions. Finally, we employed visualization tools available in TBtools (Chen et al., 2020) to generate detailed distribution maps of these *cis*-regulatory elements across each promoter region.

### 2.5 Ka and Ks calculation

To detect whether Darwinian positive selection participated in promoting gene divergence following duplication, we identified the paralogs of *SmGH3*, *AtGH3*, and *OsGH3* genes based on the phylogenetic tree. Then, we employed the PAL2NAL program (Suyama et al., 2006) to estimate the nonsynonymous (Ka) and synonymous (Ks) substitution rates, as well as the Ka/Ks ratio (nonsynonymous/synonymous substitution rate) for each paralogous gene pair. Generally, a Ka/Ks ratio of 1, greater than 1, and less than 1 indicates neutral evolution, positive selection, and negative or purifying selection, respectively (Wang et al., 2005). To further analyze selection pressures across gene regions, we calculated Ka/Ks ratios using a sliding window of 20 amino acids (Fares, 2004). Consistently, in regions where all paralogous genes showed evidence of neutral evolution, positive selection, or purifying selection, the Ka/Ks ratios consistently reflected these patterns, with ratios of 1, greater than 1, and less than 1, respectively (Fares, 2004; Wang et al., 2019).

### 2.6 Detection of positive selection

To preliminarily investigate whether the *GH3* gene family of *A. thaliana*, *S. miltiorrhiza*, and *O. sativa* exhibited evidence of positive selection (Yang et al., 2000), we examined the hypothesis of positive selection among the *GH3* genes in these species using the codeml program (Yang et al., 2000) in PAML (Phylogenetic Analysis by Maximum Likelihood) v4.9a (Nielsen and Yang, 1998; Yang, 2000, 2007) with site models and branch-site models.

In the site models, six codon substitution models M0 (one ratio), M3 (discrete), M1a (neutral), M2a (selection), M7 (beta), and M8 (beta + ω) were applied to identify codons subjected to positive selection and to detect the positively selected sites (Li et al., 2015; Wang et al., 2019). The codeml program was used to calculate the Ka/Ks ratio and to detect the variation in the *ω* parameter among sites by performing likelihood ratio tests (LRTs) between the following site model comparisons: M0 vs. M3, M1a vs. M2a, and M7 vs. M8. The detailed information on codon substitution models can refer to the previous studies (Li et al., 2015; Wang et al., 2017, 2019). Branch-site models hypothesize the different evolutionary rates to vary among different sites and branches simultaneously (Yang, 2007). We applied the improved branch-site model to compare the ratio of Ka/Ks substitution rates between branches, and to detect the positive selection amino acid sites of *SmGH3*, *AtGH3*, and *OsGH3* genes (Zhang et al., 2005). Consistent with previous methods (Li et al., 2015; Wang et al., 2017), in the branch-site model, all the branches were categorized into foreground and background groups. When the foreground branches were examined for positive selection, the other branches on the tree were used as the background. For each branch, the ratio of Ka/Ks substitution rates was calculated with the Null Model (*ω* = 1) and Alternative Model (*ω* > 1) (Li et al., 2015; Wang et al., 2017). The methods to identify the positive selection sits and estimate the Posterior probabilities (Qks) followed the previously described (Li et al., 2015; Wang et al., 2019).

### 2.7 Estimation of functional divergence

To investigate the functional divergence between subgroup genes of *SmGH3*, *AtGH3*, and *OsGH3*, we utilized the Diverge 3.0 software (Gu et al., 2013) to estimate significant changes in site-specific shifts based on maximum likelihood procedures. Then, we calculated the coefficients of Type-I and Type-II functional divergences (*θ*_I_ and *θ*_II_) between two clusters, following the previously described methods (Gu, 2006; Li et al., 2015; Wang et al., 2017, 2019). In brief, *θ*_I_ > 0 indicates site-specific alerted selective constraints, while *θ*_II_ > 0 demonstrates a radical shift in amino acid physiochemical property happened following gene duplication or speciation, respectively (Gu, 1999; Li et al., 2015; Wang et al., 2019). Detailed interpretations of *θ*_I_ and *θ*_II_, can be found in previous studies (Gu, 1999; Li et al., 2015; Wang et al., 2019). The neighbor-joining tree used for functional divergence analysis was reconstructed with the amino acid sequences of SmGH3, AtGH3, and OsGH3 under the MEGA 6.0 software (Tamura et al., 2013).

We also employed the Posterior probabilities (Qks) to identify amino acid sites associated with functional divergence. Generally, the larger Qk stands for the higher possibility that the evolutionary rate or the radical change in the amino acid property of a site that was different between the two groups (Gu, 2006; Li et al., 2015; Wang et al., 2019). Additionally, the Qk cutoff for identifying residues related to *θ*_I_ and *θ*_II_ between gene groups was determined following previously described methods (Gu, 2006; Li et al., 2015; Wang et al., 2019).

### 2.8 Expression analysis of *AtGH3*, *OsGH3* and *SmGH3* genes

For preliminary analysis of the expression patterns of *AtGH3* and *OsGH3* genes, we employed their CDS sequences as query sequences to identify EST sequences corresponding to these genes from the GenBank EST database (https://www.ncbi.nlm.nih.gov/genbank/dbest/) (Boguski et al., 1993) using the blastn suite with default parameters. The identified EST sequences were considered to correspond to the *GH3* genes if they met the following criteria: longer than 160 bp, with a threshold of less than 10^−10^, and a hit rate above 95% (Yang et al., 2008). Finally, the identified EST sequences were classified according to their tissue origin based on GENEVESTIGATOR (Zimmermann et al., 2004). Meanwhile, we analyzed the tissue-specific expression patterns of *SmGH3* genes based on the RPKM (Reads Per Kilobase per Million) values of *S. miltiorrhiza* RNA-seq data from roots, stems, leaves, and flowers tissues (SRP051524, SRP051564, SRP028388) (Zhang et al., 2015), using the Mev program (Saeed et al., 2003).

## 3 Results

### 3.1 Sequence feature of GH3 genes in A. thaliana, S. miltiorrhiza, and O. sativa

With the 19 AtGH3 and OsGH3 amino acid sequences and the basic characteristics of GH3 protein, we carefully surveyed the *S. miltiorrhiza* genome, eight members of *SmGH3* genes were identified (**Supplementary Table 1**). For the *AtGH3*, *OsGH3*, and *SmGH3* genes, the lengths ranged from 2025 bp (*AT1G48660*) to 4064 bp (*AT4G03400*), 1561 bp (*Os11g0528700*) to 8610 bp (*Os07g0671500*), and 1996 bp (*SMil_00018075*) to 4103 bp (*SMil_00006699*), respectively (**Supplementary Table 1**). The corresponding protein lengths varied from 525 aa (*AT1G48670*) to 672 aa (*AT5G13360*), 441 aa (*Os07g0576500*) to 629 aa (*Os05g0500900*), and 406 aa (*SMil_00017300*) to 624 aa (*SMil_00003673*), respectively (**Supplementary Table 1**). Furthermore, the molecular weights of the AtGH3, OsGH3, and SmGH3 proteins ranged from 64.12 kDa (*AT1G48660*) to 75.87 kDa (*AT5G13360*), 67.37 kDa (*Os01g0785400*) to 69.02 kDa (*Os06g0499500*), and 45.82 kDa (*SMil_00017300*) to 69.24 kDa (*SMil_00003673*), respectively (**Supplementary Table 1**). Additionally, the theoretical isoelectric points (*pI*) of the proteins were observed to range from 4.91 (*AT5G13320*) to 6.08 (*AT5G13360* and *AT2G47750*), 5.03 (*Os11g0528700*) to 6.87 (*Os05g0143800*), and 5.57 (*SMil_00016018*) to 7.68 (*SMil_00006699*), respectively (**Supplementary Table 1**).

### 3.2 Multiple sequence alignment analysis

Multiple sequence alignment showed that mostly members of SmGH3, AtGH3, and OsGH3 proteins contain two mainly motifs of nucleotide ATP/AMP binding motif 1 and hormone-binding motif 2 (Chang et al., 1997; Singh et al., 2014; Yu et al., 2018) (**Supplementary Figure 1**). Pairwise analyses of the SmGH3, AtGH3, and OsGH3 amino acid sequences showed that the overall similarity level ranged from as low as 25.2% between *AT5G13360* and *SMil_00017300* to a notably high similarity of 92.2% between *AT4G27260* and *AT5G54510* (**Supplementary Table 2**). High homology levels suggest they may carry out essentially similar functions, whereas low homology levels may indicate their distinct evolutionary origins and functional diversification (Kumar et al., 2012).

### 3.3 Phylogenetic relationship analysis

**Figure 1:**
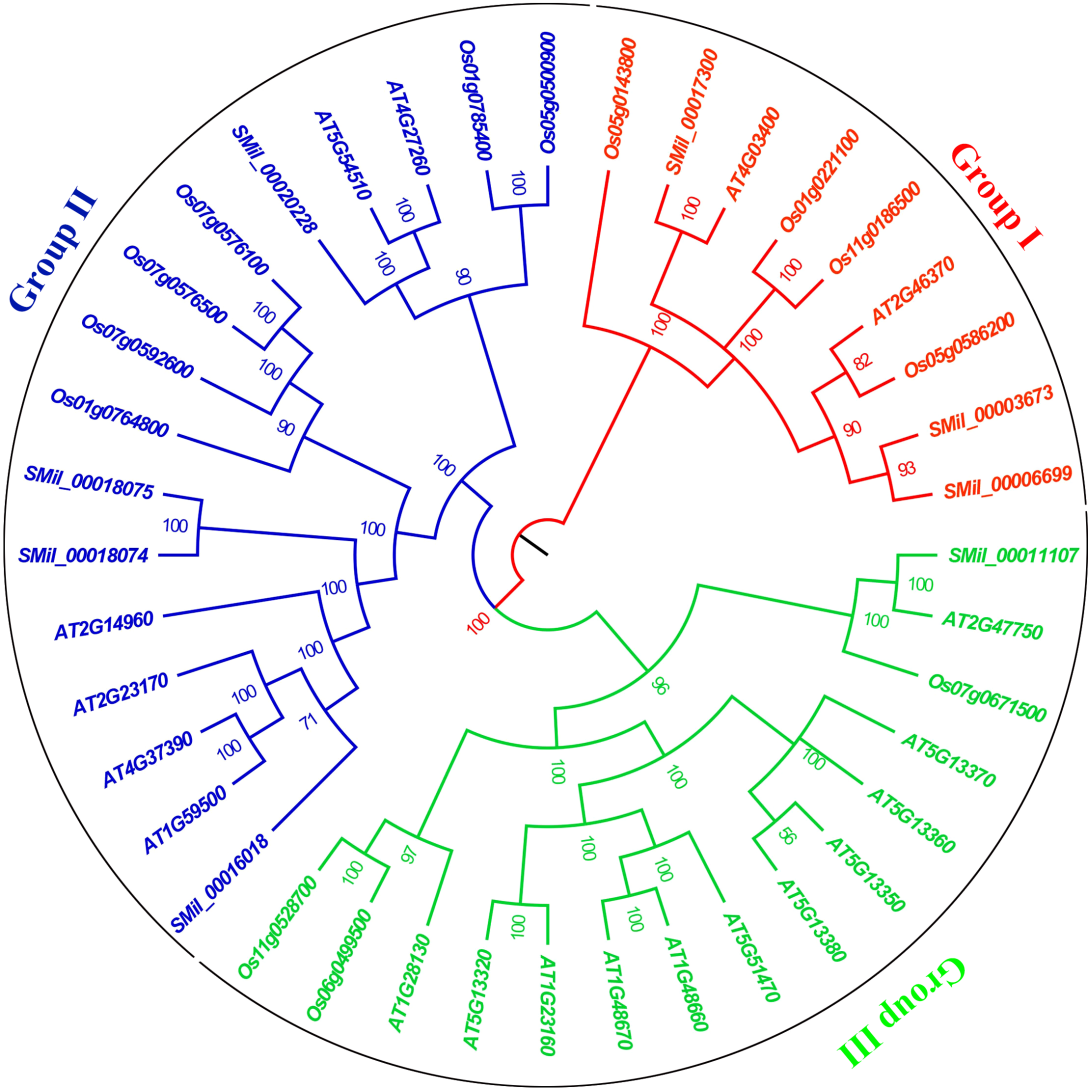
The phylogenetic tree for the *GH3* gene family in *A. thaliana*, *S. miltiorrhiza*, and *O. sativa*. The tree was constructed using Bayesian inference implemented in MrBayes based on the 40 amino acid sequences of the SmGH3, AtGH3 and OsGH3 under the model of JTT + I + G. Groups I II and III are marked with red, blue and green, respectively.

Based on sequence homology, the GH3 proteins from these species were distinctly categorized into three major groups (I, II and III), which correlated with their functions and sequence similarities (**Figure 1**). High bootstrap values for all the subgroups indicated that the genes in each subgroup might share a similar origin, which advices that the same clusters could have the same function (Staswick et al., 2002, 2005). Group I comprises nine members, with three from *S. miltiorrhiza*, two from *A. thaliana* and four from *O. sativa*. Group II contains four *SmGH3*, six *AtGH3*, and six *OsGH3 g*enes. Group III includes one *SmGH3* gene, 11 *AtGH3* genes, and three *OsGH3* genes (**Figure 1**). Additionally, paralogous gene pairs within the *SmGH3* gene family were identified, including *SMil_00018074* and *SMil_00018075* in Group II, and *SMil_00003673* and *SMil_00006699* in Group I (**Figure 1**). Within the *AtGH3* gene family, five paralogous gene pairs were discovered, including *AT1G48670* and *AT1G48660*; *AT1G23160* and *AT5G13320*; *AT5G13350* and *AT5G13380* in Group III; *AT1G59500* and *AT4G37390*; as well as *AT5G54510* and *AT4G27260* in Group II. Furthermore, in the *OsGH3* gene family, four paralogous gene pairs were found: *Os06g0499500* and *Os11g0528700* in Group III (**Figure 1**), *Os07g0576500* and *Os07g0576100*; *Os01g0785400* and *Os05g0500900* in Group II; and *Os01g0221100* and *Os11g0186500* belong to Group I (**Figure 1**).

### 3.4 Gene structure and motif analysis

**Figure 2:**
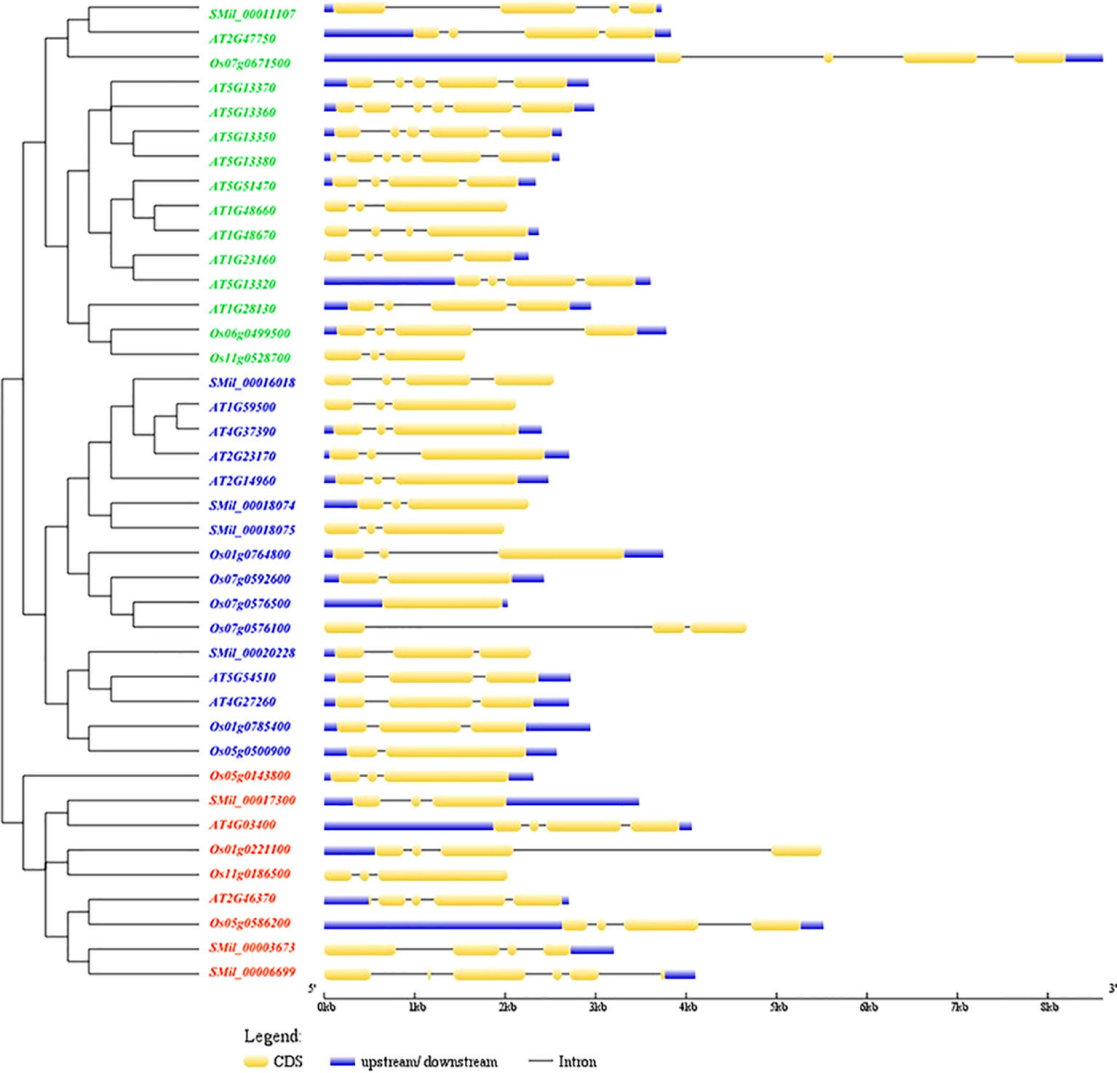
The phylogenetic tree and the structural features analysis of each *GH3* gene in *A. thaliana*, *S. miltiorrhiza*, and *O. sativa*. The exons were represented by yellow rectangles. The black lines connecting 2 exons represented introns.

Gene structure analysis revealed that the number of exons in *SmGH3*, *AtGH3*, and *OsGH3* genes ranged from one to six, and averaging three to four exons across the three species (**Figure 2**). Additionally, the number of introns varied from zero to five. Notably, a single intronless gene (*Os07g0576500*) was identified (**Figure 2**). Furthermore, the correlation between gene structure and phylogenetic tree analysis reveals that genes within the same group exhibit similar structural patterns, suggesting that genes in the same subgroup may share analogous functions (**Figure 2**).

**Figure 3:**
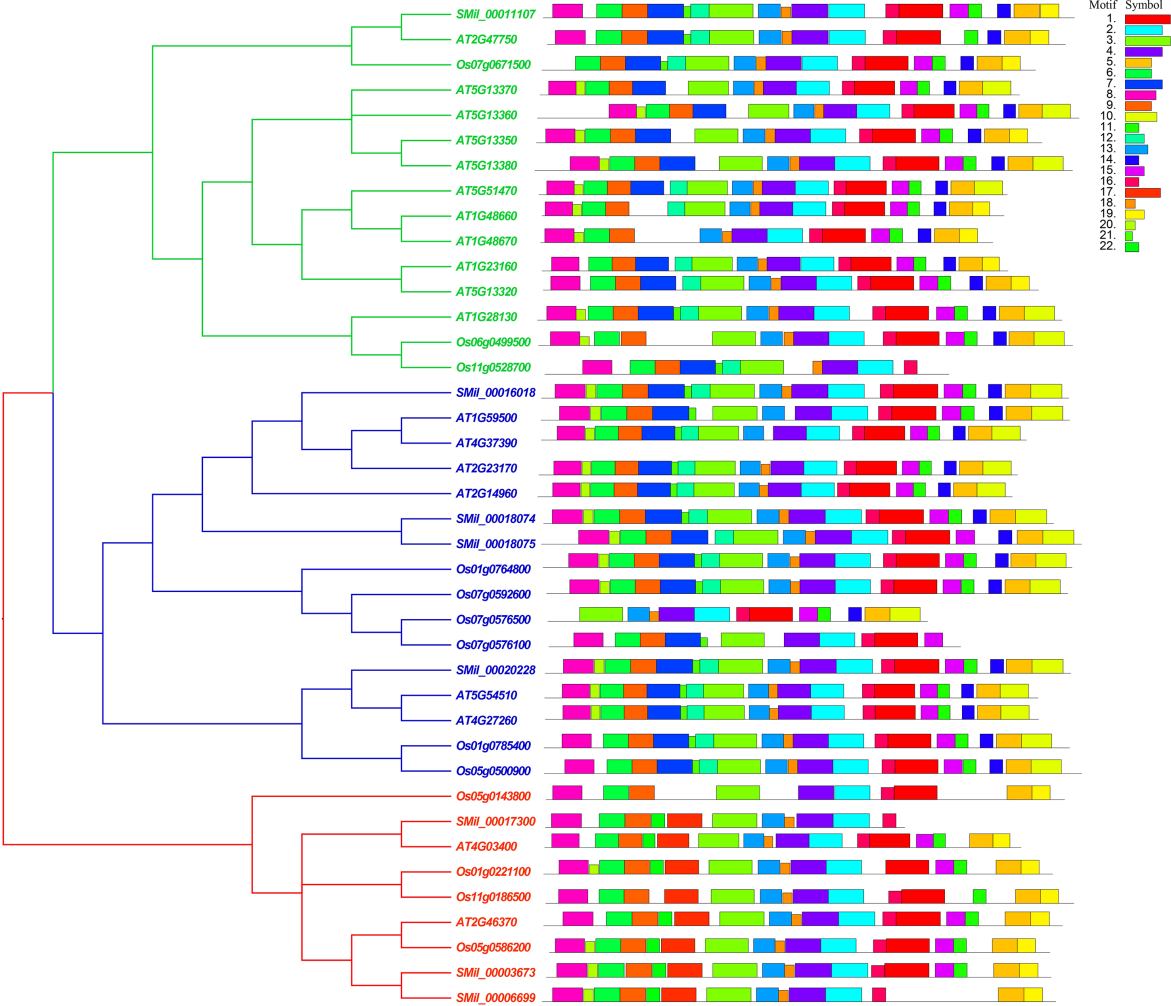
Phylogenetic tree and motif conjoint analysis of GH3 proteins in *A. thaliana*, *S. miltiorrhiza*, and *O. sativa*. Different color boxes represent different types of motifs.

With the online MEME suite, 22 conserved motifs within the amino acid sequences of AtGH3, OsGH3, and SmGH3 were identified. The normal expression sequences and diagram of these 22 motifs are listed in **Supplementary Table 3** and **4**, respectively. The results revealed that the frequencies of these motifs ranged from 7 to 40 (**Supplementary Table 3**). Additionally, the number of amino acids comprising each GH3 motif varied from 8 to 50, and the number of motifs present in each GH3 protein ranged from 11 to 19 (**Supplementary Table 3**). Moreover, among the motifs, motifs 2, 3, 4, 6, 8, 9, and 16 were commonly found in AtGH3, OsGH3, and SmGH3 proteins (**Figure 3**, **Supplementary Table 4**).

Furthermore, the integration of phylogenetic tree analysis with motif composition revealed that GH3 proteins within the same subgroup exhibited highly conserved motif arrangements and compositions (**Figure 3**), suggesting that genes within the same subgroup likely share analogous functional roles. Whereas, motifs 7, 10 and 12 were uniquely present in both Group I and Group II. Motif 19 was specifically found in Group I and Group III, while motifs 17 and 22 were exclusively present in Group III. Notably, motif 19 in Group I was uniquely identified in *A. thaliana*, and motif 14 in Group III was exclusively detected in *O. sativa* (**Figure 3**, **Supplementary Table 4**). These results suggest that group-specific sequence motifs are present in different groups, potentially contributing to functional divergence among these groups.

### 3.5 *Cis*-regulatory elements of promoter analysis in *AtGH3*, *OsGH3 and SmGH3*

**Figure 4:**
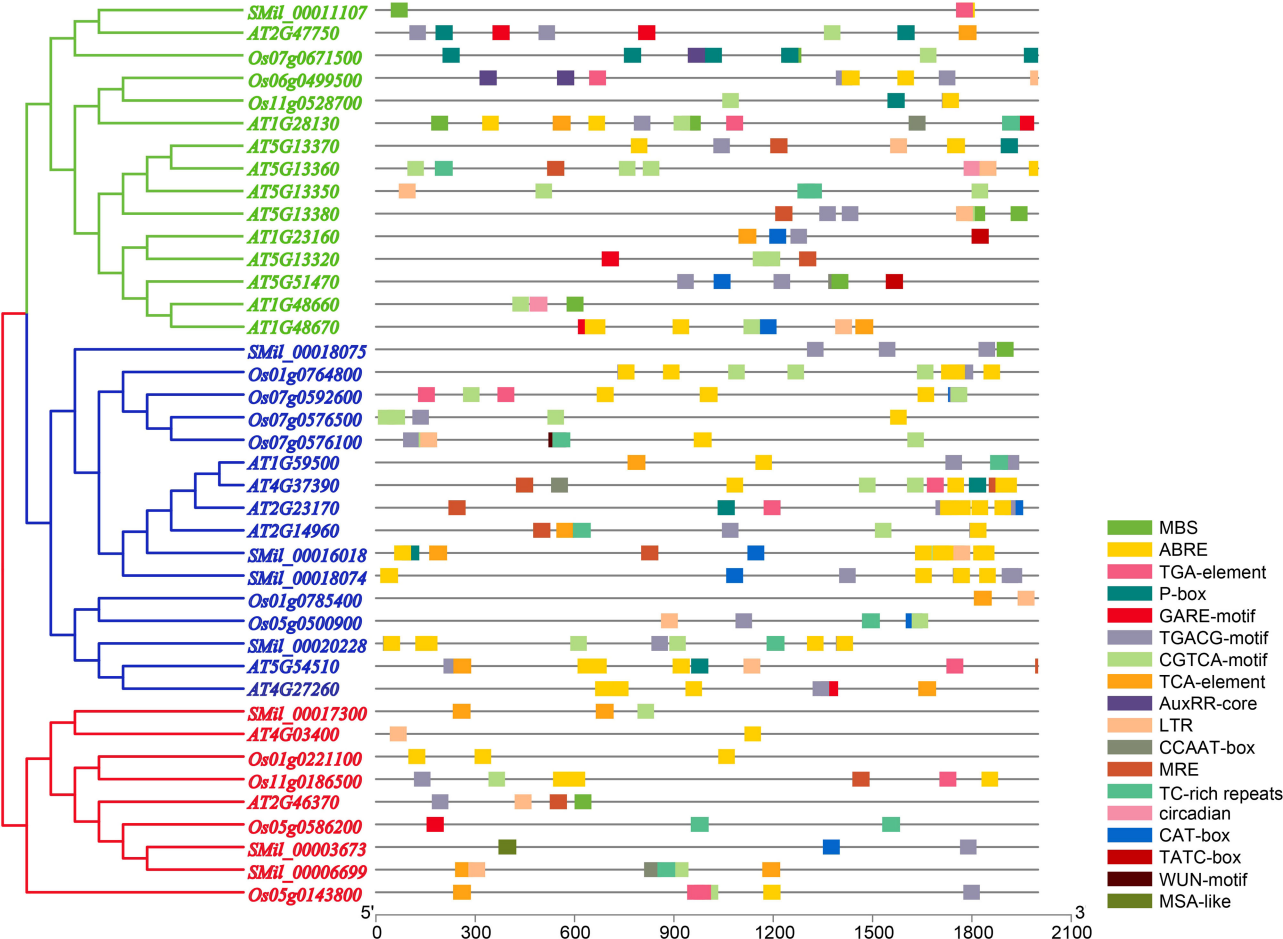
*Cis*-Element distribution analysis in putative promoters of *SmGH3*, *AtGH3* and *OsGH3* genes.

The analysis of *cis*-regulatory elements within the promoter sequences revealed that they are enriched with plant hormone response elements. These include abscisic acid-responsive elements (ABREs), which are implicated in ABA signaling; CGTCA- and TGACG-motifs, which are involved in MeJA responsiveness; and TCA-elements specifically associated with SA signaling (**Figure 4**, **Supplementary Table 5**). Specifically, nearly all *AtGH3*, *OsGH3*, and *SmGH3* gene promoters contain *cis*-regulatory elements implicated in MeJA-responsiveness (**Figure 4**, **Supplementary Table 5**). 20 *GH3* gene promoters were found to harbor *cis*-regulatory elements associated with GA responsiveness, while 24 promoters contained elements linked to ABA responsiveness (**Figure 4**, **Supplementary Table 5**). Additionally, 15 *GH3* gene promoters were associated with SA responsiveness, and 11 promoters contained auxin-responsive regulatory elements (**Figure 4**, **Supplementary Table 5**). These findings highlight the significant role of *GH3* genes in plant hormone regulation. Furthermore, 14 *GH3* gene promoters were found to contain *cis*-regulatory elements associated with low-temperature responsiveness (LRT), while 10 promoters were identified to harbor MYB binding sites (MBS) linked to drought-inducibility (**Figure 4**, **Supplementary Table 5**). Notably, one *GH3* gene promoter was found to contain a wound-responsive element (WUN-motif) (**Figure 4**, **Supplementary Table 5**). In light of these findings, it appears that the expression of the *GH3* gene is closely linked to various plant stress responses.

### 3.6 Driving forces for genetic divergence

**Figure 5:**
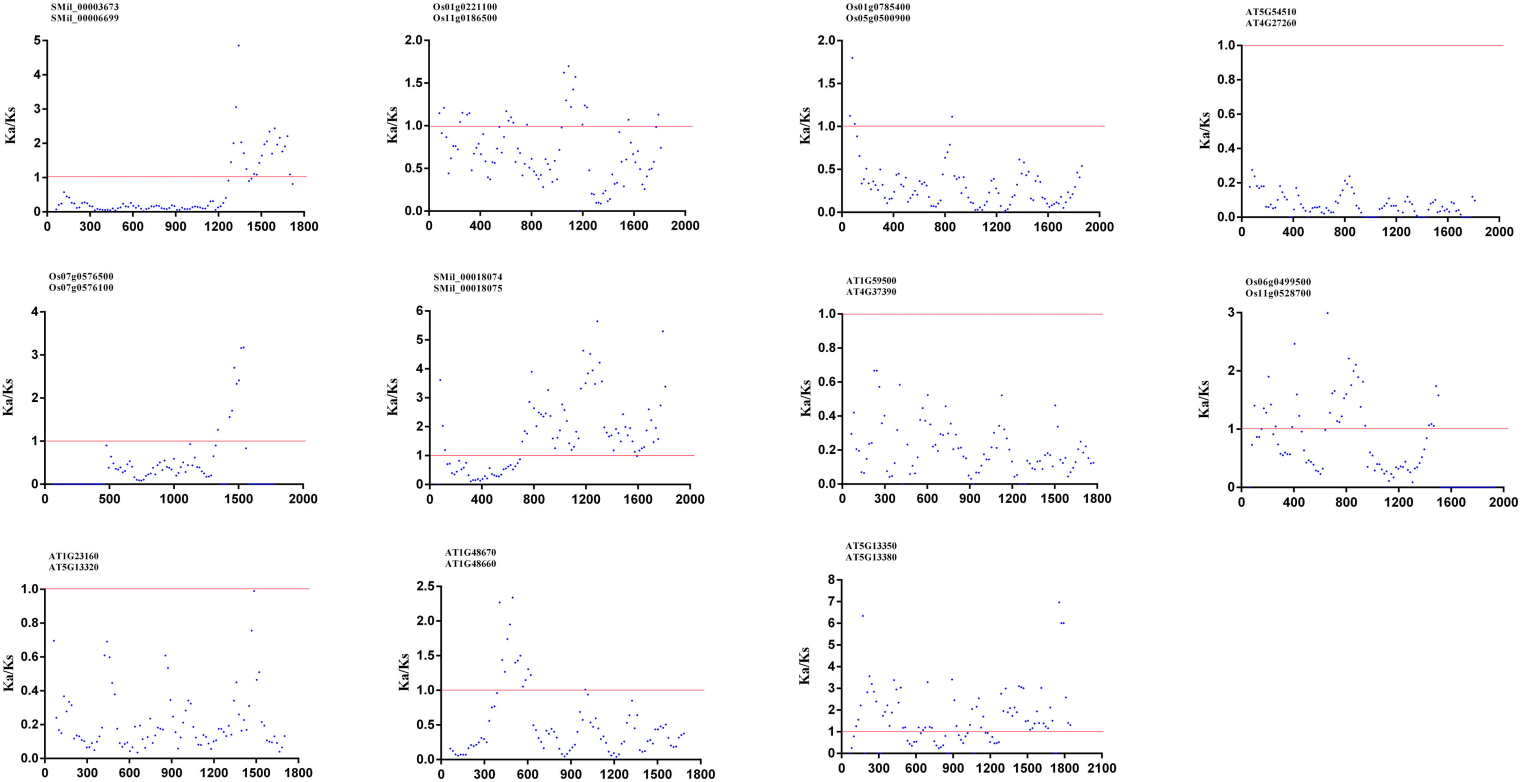
The Ka/Ks ratios for GH3–11 paralogous pairs proteins in *A. thaliana*, *S. miltiorrhiza*, and *O. sativa* with a sliding window of 20 amino acids. The plot shows the Ka/Ks ratios at various positions for the coding region of *GH3* genes.

To investigate the forces driving genetic divergence, we calculated the ratio of Ka/Ks with the coding sequences of paralogous pairs within the *AtGH3, OsGH3*, and *SmGH3* genes. Our results showed that the Ka/Ks ratios were less than 1 for five *AtGH3*, four *OsGH3*, and two *SmGH3* paralogous pairs, indicating that negative selection has acted on these genes (**Supplementary Table 6**). Meanwhile, we also calculated the Ka/Ks ratios for all the paralogous genes using a sliding window of 20 amino acids. Our results revealed that the Ka/Ks ratio was consistently less than 1 in most regions, with only a small fraction of regions showing a Ka/Ks ratio greater than 1 (**Figure 5**). Notably, two specific paralogous pairs, *AT5g13350* and *AT5g13380*, as well as *SMil_00018074* and *SMil_00018075*, exhibited a majority of their analyzed regions with Ka/Ks > 1 (**Figure 5**). Nevertheless, the overall Ka/Ks ratio for these genes remained below 1 (**Supplementary Table 6**). These findings further suggest that *GH3* genes are divergent under the purifying pressure in *A. thaliana*, *S. miltiorrhiza*, and *O. sativa*.

### 3.7 Positive selection on *AtGH3*, *OsGH3 and SmGH3* genes

To preliminarily investigate the evolutionary mechanism of *GH3* gene family in *Arabidopsis*, *S. miltiorrhiza* and *O. sativa*, we assessed the hypothesis of positive selection of *AtGH3*, *OsGH3* and *SmGH3* genes using the PAML package (Yang et al., 2000; Yang, 2007).

In the site models, following the analysis method described in the previous study (Li et al., 2015; Wang et al., 2017, 2019), we compared the M0 vs. M3 to test how Ka/Ks ratios differed among codon positions. Under the M0 model, the log-likelihood value was ι = -54531.985321, with a value of *ω* = 0.13572. In the meantime, the M3 model yielded a log-likelihood value of ι = -53113.4944310, with three *ω* estimates (*ω*_0_ = 0.01691, *ω*_1_ = 0.10134, and *ω*_2_ = 0.30575). These results suggest that relaxed purifying selection is the predominant evolutionary force acting on the *GH3* gene family in *A. thaliana*, *S. miltiorrhiza* and *O. sativa* (**Table 1**). Furthermore, the twice log likelihood difference (2ΔlnL) between M3 and M0 was calculated as 2836.98178 (**Table 1**), which was strongly statistically significant (*p* < 0.01) and suggested that M3 was better than M0. Therefore, the results indicated that different sites bear different selection pressures and also revealed fluctuations in the overall level of selective constraints (Li et al., 2015; Wang et al., 2017, 2019). Next, the M2a vs. M1a and M8 vs. M7 were compared to test whether positive selection promoted divergence between genes (Li et al., 2015). The log-likelihood values for M1a and M2a were ι= -54101.460731 and ι= -54101.460957, respectively. Similarly, under models M7 and M8, the log-likelihood values were ι= -53553.845867 and ι= -53550.001494, respectively (**Table 1**). The 2ΔlnL values for M2a vs. M1a and M8 vs. M7 were 0 and 7.39, respectively. Thus, in both comparisons, there was no statistical significance, and no site was detected under positive selection at the level of 95% (**Table 1**). All parameter estimates are presented in **Table 1**.

**Table 1:** Tests for positive selection among codons of *SmGH3*, *AtGH3* and *OsGH3* genes using site models.

Model	np	lnL	Estimates of parameter^1^		2ΔlnL	Positive selection sites^2^
			Frequency	dN/dS		
M0 (one-ratio)	80	-54531.985321		0.13572		None
M3 (discrete)	84	-53113.494431	*p*_0_ = 0.18365 *p*_1_ = 0.41779 *p*_2_ = 0.39856	ω_0_ = 0.01691 ω_1_ = 0.10134 ω_2_ = 0.30575	2836.98178(M3 vs. M0)**	Not allowed
M1a (nearly neutral)	81	-54101.460731	*p*_0_ = 0.82037 *p*_1_ = 0.17963	ω_0_ = 0.13449 ω_1_ = 1.00000		None
M2a (positive selection)	83	-54101.460957	*p*_0_ = 0.82037 *p*_1_ = 0.17963 *p*_2_ = 0.00000	ω_0_ = 0.13449 ω_1_ = 1.00000 ω_2_ = 318.98328	0 (M2a vs. M1a)	Not allowed
M7 (beta)	81	-53553.845867	*p* = 0.87388 *q* = 3.76068			None
M8 (beta & ω)	83	-53550.001494	*p*_0_ = 0.97816 *p* = 0.90568 *q* = 4.17138 *p*_1_ = 0.02184	ω = 1.90498	7.392934 (M8 vs. M7) *	584 A

*p < 0.05 and **p < 0.01 (x^2^ test).

^1^ω was estimated under model M0, M3, M7, and M8; *p* and *q* are the parameters of the beta distribution.

^2^The number of amino acid sites estimated to have undergone positive selection.

In branch-site models, we compared the Null and Alternative models to test the positive sites under positive selection in particular lineage groups. The results showed that the two models differed significantly (*p* < 0.01) when each lineage group was designated as the foreground branch. This suggests that the *GH3* lineage groups from *A. thaliana*, *S. miltiorrhiza*, and *O. sativa* exhibit distinct evolutionary rates (**Table 2**). Additionally, when the foreground branch was assigned to group I, a total of 570 positively selected sites were found, with a 2ΔlnL value of 38.546436 (*p* < 0.01) between Null and Alternative models. When the foreground branch was set to group II, 13 positively selected sites were identified, yielding a 2ΔlnL value of 12.33403 (*p* < 0.01). Similarly, when the foreground branch was assigned to group III, 18 positively selected sites were detected, with a 2ΔlnL value of 15.767194 (*p* < 0.01). These results indicate that all three lineage groups were under positive selection (*p* < 0.01). However, no site was positively selected at a level of 95% (**Table 2**). Furthermore, the remarkably higher number of positively selected sites in group I suggests that this group may be undergoing strong positive Darwinian selection compared to the other two groups. All parameter estimates are presented in **Table 2**.

**Table 2:** Selective pressure analyses of *SmGH3*, *AtGH3* and *OsGH3* genes using branch-site models.

Foreground branches	Branch-site model	lnL	2ΔlnL	P Value	ω Values^1^	Positively selected sites^2^
Group I	Null	-54569.474808	38.546436	< 0.01	ω_0_ = 0.13548 ω_1_ = 1.00000 ω_2_ = 1.00000	570 Sites were detected
	Alternative	-54550.201590			ω_0_ = 0.13670 ω_1_ = 1.00000 ω_2_ = 999.00000	
Group II	Null	-54589.735993	12.33403	< 0.01	ω_0_ = 0.13470 ω_1_ = 1.00000 ω_2_ = 1.00000	13 Sites were detected
	Alternative	-54583.568978			ω_0_ = 0.13475 ω_1_ = 1.00000 ω_2_ = 248.12040	
Group III	Null	-54587.802687	15.767194	< 0.01	ω_0_ = 0.13418 ω_1_ = 1.00000 ω_2_ = 1.00000	18 Sites were detected
	Alternative	-54579.919090			ω_0_ = 0.13443 ω_1_ = 1.00000 ω_2_ = 21.24914	

*p < 0.05 and **p < 0.01 (x^2^ test).

^1^ω was estimated under model Null and Alternative.

^2^The number of amino acid sites estimated to have undergone positive selection.

### 3.8 Functional divergence analysis of SmGH3, AtGH3 and OsGH3 proteins

Based on the neighbor-joining tree, the *GH3* gene family in *A. thaliana*, *S. miltiorrhiza* and *O. sativa* were also divided into three primary clusters (**Supplementary Figure 2**). Using the DIVERGE program, we evaluated the rates of evolutionary shift and the properties of altered amino acids following gene duplication (Gu, 1999; 2006).

The results revealed that the coefficients for the Type-I functional divergence (*θ*_I)_ among Group I vs. Group II, Group I vs. Group III, and Group II vs. Group III were 0.561910, 0.440390 and 0.237761, respectively (**Supplementary Table 7**). Notably, all *θ*_I_ coefficients were greater than 0 across all group pairs. This suggested that some amino acid sites may have undergone significant site-specific changes between these group pairs, which bring about a subgroup-specific functional divergence during their evolution (Gu, 2006; Gu et al., 2013; Wang et al., 2017). Furthermore, the results also revealed that *θ*_I_ values of Group I vs. Group II, Group I vs. Group III, and Group II vs. Group III were statistically significant (*p* < 0.01) (**Supplementary Table 7**). This observation is consistent with our phylogenetic analysis that functional divergence has occurred among members of different groups. In addition, the coefficients of Type-II functional divergence (*θ*_II_) for all three pairs were less than 0 (*θ*_II_ = -0.136874, *θ*_II_ = -0.637158 and *θ*_II_ = -0.283415), and not statistically significant among the three group pairs (*p* = 0.2514, *p* = 0.02275 and *p* = 0.08691, respectively) (**Supplementary Table 8**). Those results suggest that most amino acids of SmGH3, AtGH3, and OsGH3 proteins have not undergone significant changes in their physical and chemical properties (Li et al., 2015; Wang et al., 2017, 2019).

**Figure 6:**
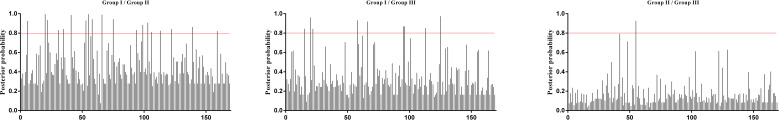
Site-specific prediction for type-I functional divergence between groups of *SmGH3*, *AtGH3* and *OsGH3*. The X-axis represents locations of sites. The Y-axis represents the probability of each group. The red line indicates cutoff = 0.80.

**Figure 7:**
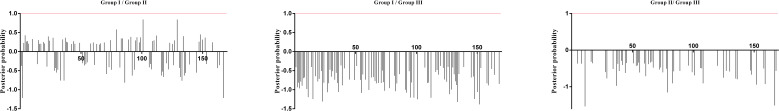
Site-specific profile for predicting critical amino acid residues responsible for the type-II functional divergence between groups of *SmGH3*, *AtGH3* and *OsGH3*. The X-axis represents locations of sites. The Y-axis represents the probability of each group. The red line indicates cutoff = 1.0.

Additionally, the positive selection sites that influencing functional divergence between the groups was also tested using the Posterior probability (Qk). As previously described (Li et al., 2015; Wang et al., 2017, 2019), we established Qk > 0.8 and 1.0 as the threshold for identifying the Type-I and Type-II functional divergence-related positive selection sites between groups, respectively. The analysis of Qks revealed that the distribution and the number of positive selection sites associated with functional divergence varied across group pairs. For Type-I functional divergence, when Qk > 0.8, all of the three clusters pairs contained positive selection sites (**Figure 6**). In contrast, for Type-II functional divergence, there was no group pair contained positive selection sites, when Qk > 1.0 (**Figure 7**). These results suggest that the identified positive selection sites probably play a crucial role in the functional divergence of *SmGH3*, *AtGH3*, and *OsGH3* during their evolutionary process. The detailed distribution patterns of positive selection sites influencing Type-I and Type-II functional divergence between groups are illustrated in **Figure 6** and **7**, **Supplementary Tables 7** and **8**.

### 3.9 The expression analysis of *AtGH3*, *OsGH3* and *SmGH3* genes

According to GENEVESTIGATOR, in *A. thaliana* and *O. sativa*, the ESTs primarily contain six tissue categories including Callus Cell, Suspension, Seedling, Inflorescence, Rosette, and Roots (Zimmermann et al., 2004). To preliminarily detect the tissue-specific expression of *AtGH3* and *OsGH3* genes, the CDS sequences of each *GH3* gene were employed to search the *A. thaliana* and *O. sativa* ESTs databases with the blastn program. Because of the specially temporal and special expression pattern for genes, and the EST database may not contain the EST resource for certain genes (Yang et al., 2008), there were five *AtGH3* genes and three *OsGH3* genes that didn’t find evidence of the expression (**Supplementary Table 9**, **10**). In *A. thaliana*, most *AtGH3* genes are predominantly expressed in tissues of Rosette, Root, and Inflorescence (**Supplementary Table 9**). Meanwhile, in *O. sativa*, most *OsGH3* genes are primarily expressed in tissues of Seedling, Inflorescence, and Callus tissues (**Supplementary Table 10**).

Based on the RPKM values derived from RNA-seq data for *S. miltiorrhiza* organs of root, stem, leaf and flower, we observed distinct expression patterns of these genes in different organs. Among these genes, *SMil_00016018*, *SMil_00018075*, and *SMil_00018074* exhibited relatively low expression levels across all examined organs (**Supplementary Figure 3**, **Supplementary Table 11**). Specifically, *SMil_00016018* and *SMil_00018075* exhibited tissue-specific expression patterns, being detectable only in the flowers and roots, while *SMil_00018074* showed no expression in the leaves. In contrast, *SMil_00006699* demonstrated relatively high expression levels across all four organs examined (**Supplementary Figure 3**, **Supplementary Table 11**). Additionally, *SMil_00003673* and *SMil_00011107* exhibited elevated expression levels in stems, whereas *SMil_00017300* showed higher expression in flowers and leaves. Notably, *SMil_00020228* demonstrated particularly high expression in roots (**Supplementary Figure 3**, **Supplementary Table 11**).

## 4 Discussion

The Gretchen Hagen 3 (GH3) proteins, a small multi-gene family within the acyl-adenylate/thioester-forming enzyme superfamily, play a critical role in regulating hormone homeostasis in plants (Baranwal et al., 2017; Kong et al., 2019). These proteins function by catalyzing the formation of amino acid conjugates, which in turn modulate various physiological processes (Kong et al., 2019). Consequently, the substrates and enzymatic products of GH3 proteins exert significant influence on plant development, growth regulation, and responses to environmental stress (Kong et al., 2019). As research progresses, an increasing number of *GH3* gene families have been identified in diverse plant species, including mosses and angiosperms (Singh et al., 2014). Through in-depth studies of this gene family, the functions of *GH3* genes in plant growth processes can be elucidated, while also allowing us to investigate the evolutionary relationships among different species.

The protein lengths of *GH3* family varied from 525 to 672 amino acids in *A. thaliana* (Staswick et al., 2002), 441 to 629 amino acids in *O. sativa* (Jain et al., 2006a), 547 to 651 amino acids in maize (Feng et al., 2015), 578 to 613 amino acids in grape (Bottcher et al., 2011), 495 to 843 amino acids in tomato (Kumar et al., 2012), 571 to 614 amino acids in apple, 542 to 644 amino acids in cotton (Yu et al., 2018), and 588 to 640 amino acids in melon (Chen et al., 2023). This variation in protein lengths suggests that *GH3* family members generally exhibit proteins longer than 400 amino acids. In this study, we identified eight *GH3* genes in the genome of *S. miltiorrhiza* based on the sequence information of *GH3* members from *A. thaliana* and *O. sativa* (Staswick et al., 2002; Jain et al., 2006b) and the reported protein lengths of *GH3* genes. The *GH3* family in *S. miltiorrhiza* is smaller than those in *A. thaliana* (19 members), *O. sativa* (13 members) and maize (13 members), but its size is comparable to that of grape (9 members). Furthermore, the parameters of SmGH3 proteins displayed high similarity to those of *GH3* family in *A. thaliana*, *O. sativa*, maize and grape, suggesting that different GH3 proteins may perform analogous functions under varying microenvironmental conditions (Feng et al., 2015). The relatively high amino acid identity of *SmGH3* genes (**Supplementary Figure 1**) also further indicates that these genes likely originated from a common ancestral sequence (Feng et al., 2015).

Gene duplications play a crucial role in gene family expansion, and Darwinian positive selection drives the expansion of gene families following duplication (Lynch and Conery, 2000). Phylogenetic analyses are essential for identifying putative paralogs, which are critical for detecting gene duplication events within gene families (Yang et al., 2008). Based on the phylogenetic tree, five pairs of paralogous genes were identified in *A. thaliana*, four pairs in *O. sativa*, and two pairs in *S. miltiorrhiza* (**Figure 1**). The findings revealed that a majority of *AtGH3*, *OsGH3*, and *SmGH3* genes are organized as paralogous pairs (53% for *A. thaliana*, 62% for *O. sativa*, and 50% for *S. miltiorrhiza*). This indicates that over half of the *GH3 g*enes in these species underwent duplication events, suggesting that *AtGH3*, *OsGH3* and *SmGH3* genes may have experienced gene family expansion following duplication during evolution (Wang et al., 2019). Additionally, tow sister pair genes (*SMil_00017300*/*AT4G03400* and *SMil_00011107*/*AT2G47750*) within the *GH3* gene family between *S. miltiorrhiza* and *A. thaliana* were identified as ortholog genes with a high bootstrap value of 100%. No sister gene pairs could be identified as orthologous between *S. miltiorrhiza* and *O. sativa* within the *GH3* gene family (**Figure 1**). Notably, the phylogenetic relationship between *S. miltiorrhiza* and *A. thaliana* is significantly closer than that between *S. miltiorrhiza* and *O. sativa*. Our findings suggest that the functions of *SmGH3* genes may share similarities with *GH3* genes in *A. thaliana*.

*Cis*-regulatory elements are essential for gene transcription and expression, playing a crucial role in regulating plant adaptability to diverse environmental conditions through various molecular mechanisms (Liu et al., 2016; Yao et al., 2023). By investigating the multitude of *cis*-regulatory elements in the promoter regions of *AtGH3*, *OsGH3* and *SmGH3* genes, we could gain preliminary insights into the diverse functions of *GH3* genes in plants growth and development. In the present study, we found that *cis*-regulatory elements associated with plant hormone response were significantly represented among the promoter regions of *AtGH3*, *OsGH3* and *SmGH3* genes (**Figure 4**, **Supplementary Table 5**). This finding suggested that the expression of *GH3* genes may be modulated through multiple plant hormone pathways. Additionally, the promoters of *AtGH3*, *OsGH3* and *SmGH3* genes were found to contain numerous stress-related regulatory elements, which was consistent with previous study that *GH3* genes are widely involved in disease resistance and biotic and abiotic stress responses (Jain and Khurana, 2009; Feng et al., 2015; Ai et al., 2023).

Our analysis of the Ka/Ks ratios for *AtGH3*, *OsGH3* and *SmGH3* paralogs revealed that all these ratios were less than 1 (**Supplementary Table 6**). Moreover, the sliding window analysis indicated that regions exhibiting Ka/Ks ratios greater than 1 across all paralogous genes were relatively rare (**Figure 5**). These results collectively suggest that *GH3* genes in these species have likely been subject to negative selection, which play a dominant role in driving force the gene divergence. This finding is consistent with previous studies on wheat, Oryza species, and Rosaceae species (Kong et al., 2019; Jiang et al., 2020; Guo et al., 2025). Additionally, site models analysis demonstrated that different sites within the *GH3* gene family are subject to varying selection pressures, and purifying selection has also been a dominant force in the evolution of the *GH3* gene family in *S. miltiorrhiza*, *A. thaliana* and *O. sativ*a. Furthermore, although most positive sites were identified during the branch-site model analysis, none achieved statistical significance (*p* < 0.05) (**Table 2**). These results indicate that despite exposure to positive Darwinian selection across all subgroups and sites, the *GH3* gene family in *A. thaliana*, *S. miltiorrhiza* and *O. sativa* primarily underwent neutral evolution and negative selection. All those findings collectively provided further evidence to support our driving force analysis for the gene divergence.

In accordance with prior research, *GH3* genes are classified into three distinct groups (I, II, and III), which are characterized by significant functional divergence among their members (Staswick et al., 2002, 2005; Okrent et al., 2009). Our analysis of adaptive evolution revealed that group I has experienced strong positive Darwinian selection relative to the other two groups, likely reflecting the potential functional roles of its constituent genes. Group I GH3 proteins are primarily characterized as JA- or SA-amido synthetases, catalyzing the conversion of these hormones into their respective conjugated forms, such as JA-Ile and SA-amides (Staswick et al., 2002). JA and JA-Ile function as critical hormonal signaling molecules, regulating plant secondary metabolism, growth, defense, and development (Pieterse et al., 1998; Chehab et al., 2012; Pingping et al., 2017). Notably, most *GH3* genes are up-regulated by JA treatment (Feng et al., 2015). SA, a key phytohormone, plays a pivotal role in plant immunity defense (Gao et al., 2014) and serves as a major stress-related hormone involved in plant growth and development under abiotic stresses (Xiong et al., 2002). Consequently, the execution of these defensive functions may necessitate that *GH3* genes experience stronger positive selective pressure to enhance their adaptive potential to environmental challenges.

Motif analysis demonstrated that GH3 proteins from different subgroups exhibit distinct motif compositions and arrangements (**Supplementary Table 4**), which are indicative of specific amino acid residue changes that may contribute to functional divergence among these subgroups. Using the DIVERGE program, we conducted an in-depth analysis of the molecular mechanisms underlying this functional divergence. The results further demonstrated that specific amino acid residues underwent site-specific changes that contributed to functional divergence among *GH3* ubgroup genes in *S. miltiorrhiza*, *A. thaliana* and *O. sativ*a throughout the course of their evolution.

The tissue-specific expression of *AtGH3*, *OsGH3* and *SmGH3* genes exhibit differential expression across various tissues, potentially indicating distinct functional roles in different tissues. Similar to other species (Jain and Khurana, 2009; Feng et al., 2015), *SmGH3* exhibited tissue-specific expression patterns in *S. miltiorrhiza*. Based on the RPKM values, most *SmGH3* displayed relatively lower expression levels in the leaves compared to other organs (**Supplementary Figure 3**, **Supplementary Table 11**). These findings are consistent with previous studies on tissue-specific expression of *GH3* genes in other species, such as maize (Feng et al., 2015), tomato (Kumar et al., 2012), and apple (Yuan et al., 2013). Notably, most *SmGH3* genes were relatively highly expressed in stems, suggesting their potential roles in stem growth and development rather than in leaves.

Tissue-specific expression analysis revealed that *SMil_00020228* exhibits particularly high expression in roots (**Supplementary Figure 3**, **Supplementary Table 11**). Phylogenetic analysis demonstrated that *SMil_00020228* clusters with *SiGH3.15* (**Supplementary Figure 4**), which regulates lateral root development through modulation of auxin homeostasis in tomato (Ai et al., 2023). Moreover, the pharmacologically active components of *S. miltiorrhiza* are primarily localized in its roots, and the dried roots of *S. miltiorrhiza* have been used widely to treat various cardiovascular diseases (Wang et al., 2007; Kai et al., 2011). We propose that *SMil_00020228* likely plays a significant role not only in root development but also in the accumulation of pharmacologically active compounds in *S. miltiorrhiza*. Additionally, *AtGH3.9* (*AT2G4775*) was involved in cross talk between auxin and JA signal transduction pathways by conjugating amino acids to both methyl jasmonate and auxin, respectively (Khan and Stone, 2007; Feng et al., 2015). Similarly, in maize, *ZmGH3.9* is strongly induced by JA treatment in leaves (Feng et al., 2015). In *S. miltiorrhiza*, the closest homolog to both *AtGH3.9* and *ZmGH3.9* is *SMil_00011107* (**Figure 1**, **Supplementary Figure 4**). Based on this homology, we hypothesize that *SMil_00011107* functions as a jasmonate-responsive gene, playing a crucial role in regulating the JA signaling pathway within *S. miltiorrhiza*. Furthermore, phylogenetic tree reveals that *SMil_00016018* clusters with *SiGH3.4*, a gene that is strongly activated in the Arbuscular Mycorrhizal fungal-colonized roots, as demonstrated by previous studies in tomato (Liao et al., 2015; Chen et al., 2021). Moreover, as shown our tissue-specific expression analysis, *SMil_00016018* exhibited relatively low expression levels across all examined organs. Interestingly, this expression pattern closely resembles that of *SiGH3.4* in tomato (Liao et al., 2015). Based on these observations, we hypothesize that *SMil_00016018* may play a role analogous to *SiGH3.4* in mycorrhizal symbiosis within *S. miltiorrhiza*.

JAR1 (Jasmonic acid resistant 1) a key enzyme in jasmonate biosynthesis, is central to activating JA signaling and regulating defense and stress responses in plants (Suza and Staswick, 2008; Mosblech et al., 2011). Recently, *GH3.10* has emerged as another critical catalyst for JA-Ile synthesis, complementing the role of JAR1 (Delfin et al., 2022; Ni et al., 2025). Evolutionary analysis reveals that *SMil_00017300* clusters closely with *AtGH3.10* (*AT4G03400*) and *SiGH3.10* (Kumar et al., 2012) (**Figure 1**, **Supplementary Figure 4**). Based on these phylogenetic relationships, we hypothesize that *SMil_00017300* may play a similar functional role to *JAR1* in regulating JA signaling and defense mechanisms in *S. miltiorrhiza*. Indeed, *SMil_00017300* catalyzes the conversion of JA to JA-Ile, a biosynthetic step analogous to JAR1 activity (Shimizu et al., 2013; Delfin et al., 2022; Ni et al., 2025). JA-Ile signaling leads to the degradation of Jasmonate ZIM-domain (JAZ) proteins (Fonseca et al., 2009; Kramell et al., 2009), thereby releasing transcription factors such as MYB and MYC for activation (Ding et al., 2017; Shasha et al., 2018). Notably, both phenolic acids and tanshinones—signature bioactive compounds in *S. miltiorrhiza*—are regulated by JA signaling (Xiao et al., 2009; Wang et al., 2016) and their biosynthesis is directly influenced by MYB and MYC transcription factors (Zhang et al., 2014). Consequently, *SmGH3* genes exert regulatory control over endogenous hormone homeostasis in *S. miltiorrhiza*, which in turn modulates downstream signaling cascades, and subsequently influencing the biosynthesis of pharmacologically active components.

## 5 Conclusion

In this study, we characterized the *GH3* gene family in *S*. *miltiorrhiza* and performed a comparative analysis of the *GH3* gene family across three plant species: *S. miltiorrhiza*, *A. thaliana* and *O. sativ*a. Our analysis integrated multiple approaches, including phylogenetic tree construction, GH3 domain characterization, gene structure and conserved motif identification, *cis*-regulatory elements examination, selective constraint analysis, functional divergence analysis, and expression profiling. Phylogenetic analysis revealed that the 40 *AtGH3*, *OsGH3* and *SmGH3* genes could be clustered into three distinct subgroups. Genetic divergence analyses indicated that the *GH3* genes from *S. miltiorrhiza*, *A. thaliana*, and *O. sativa* have been subjected to purifying selection pressure. Positive selection and functional divergence analyses further demonstrated that these *GH3* genes have undergone purifying selective pressure and have diverged in their functional roles. Based on an integrated analysis of phylogenetic evolution and tissue-specific expression patterns, our findings suggest that certain members of the *SmGH3* gene family are involved in responding to the jasmonates signaling pathway, thereby playing critical roles in the biosynthesis and accumulation of pharmacologically active compounds, as well as in secondary metabolism in *S. miltiorrhiza*. Collectively, these results provide a comprehensive understanding of the *GH3* gene family, offering valuable insights for future functional studies of *GH3* genes in plants.

## Data Availability

The datasets presented in this study can be found in online repositories. The names of the repository/repositories and accession number(s) can be found in the article/supplementary material.

## Supplementary material

Supplementary Figure 1 The multiple sequence alignments of the SmGH3, AtGH3 and OsGH3 protein conserved domain, including the nucleotide (ATP/AMP) and hormone-binding motif 1 and hormone-binding motif 2.

Supplementary Figure 2 The neighbor-joining phylogenetic tree was reconstructed with GH3 amino acid sequences of *A. thaliana*, *S. miltiorrhiza*, and *O. sativa* using MEGA 6.0.

Supplementary Figure 3 Heatmaps representing the expression profiles of *SmGH3*, genes in the Flower, Leaf, Root and Stem.

Supplementary Figure 4 The phylogenetic tree for the *GH3* gene family in maize (*Zea mays*), tomato (*Solanum lycopersicum*), and *S. miltiorrhiza*.
