# Supplementary figures and images for: Genome-wide comprehensive analysis the molecular phylogenetic evolution, functional divergence and tissue-specific expression of GH3 gene family in Salvia miltiorrhiza, Arabidopsis thaliana, and Oryza sativa
Source: Front Plant Sci. 2025 Nov 14;16:1644853. doi: 10.3389/fpls.2025.1644853 (PMC12661205; doi:10.3389/fpls.2025.1644853)

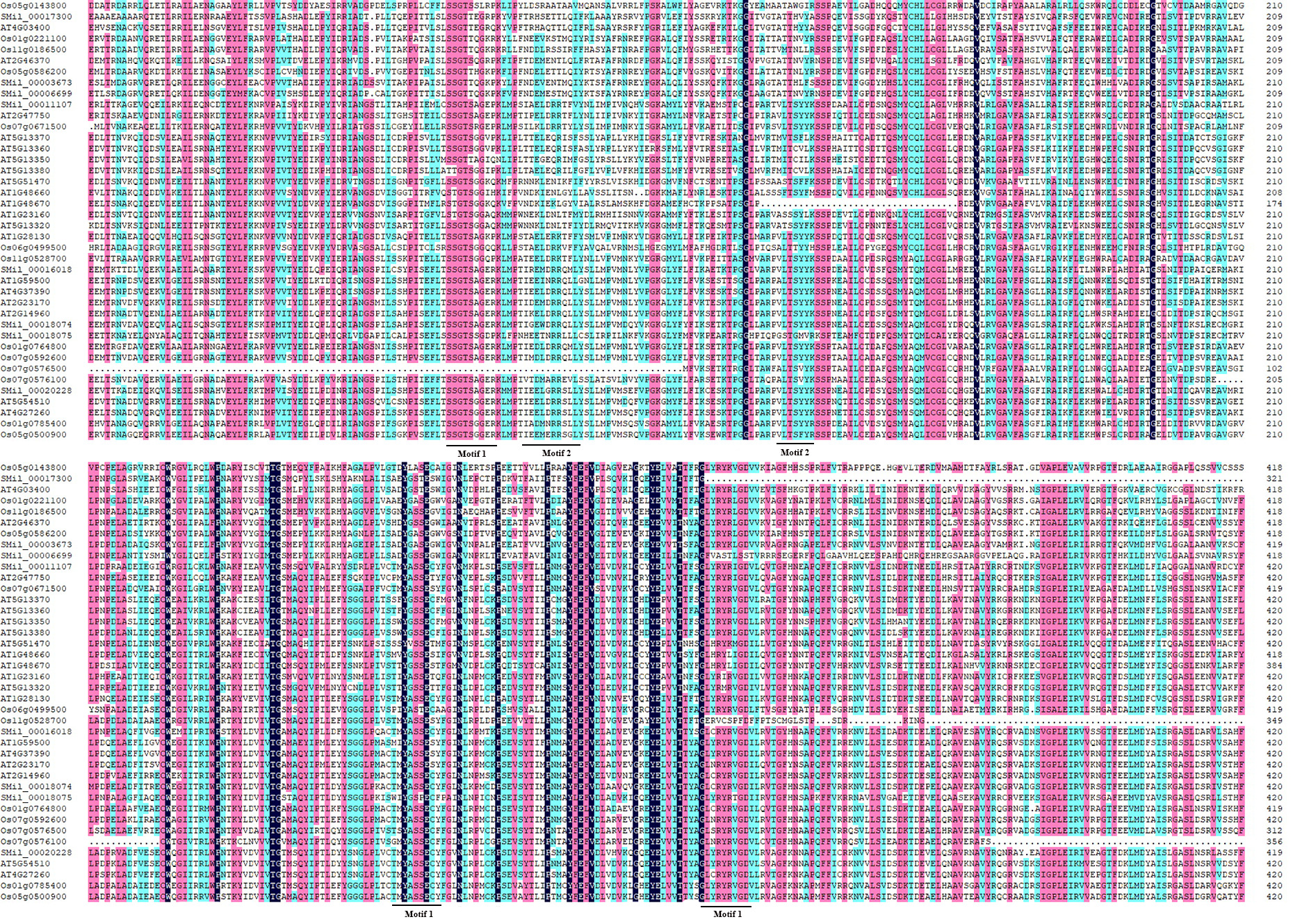

Supplement: Supplementary Figure 1 — The multiple sequence alignments of the SmGH3, AtGH3 and OsGH3 protein conserved domain, including the nucleotide (ATP/AMP) and hormone-binding motif 1 and hormone-binding motif 2. [file Image1.tif]

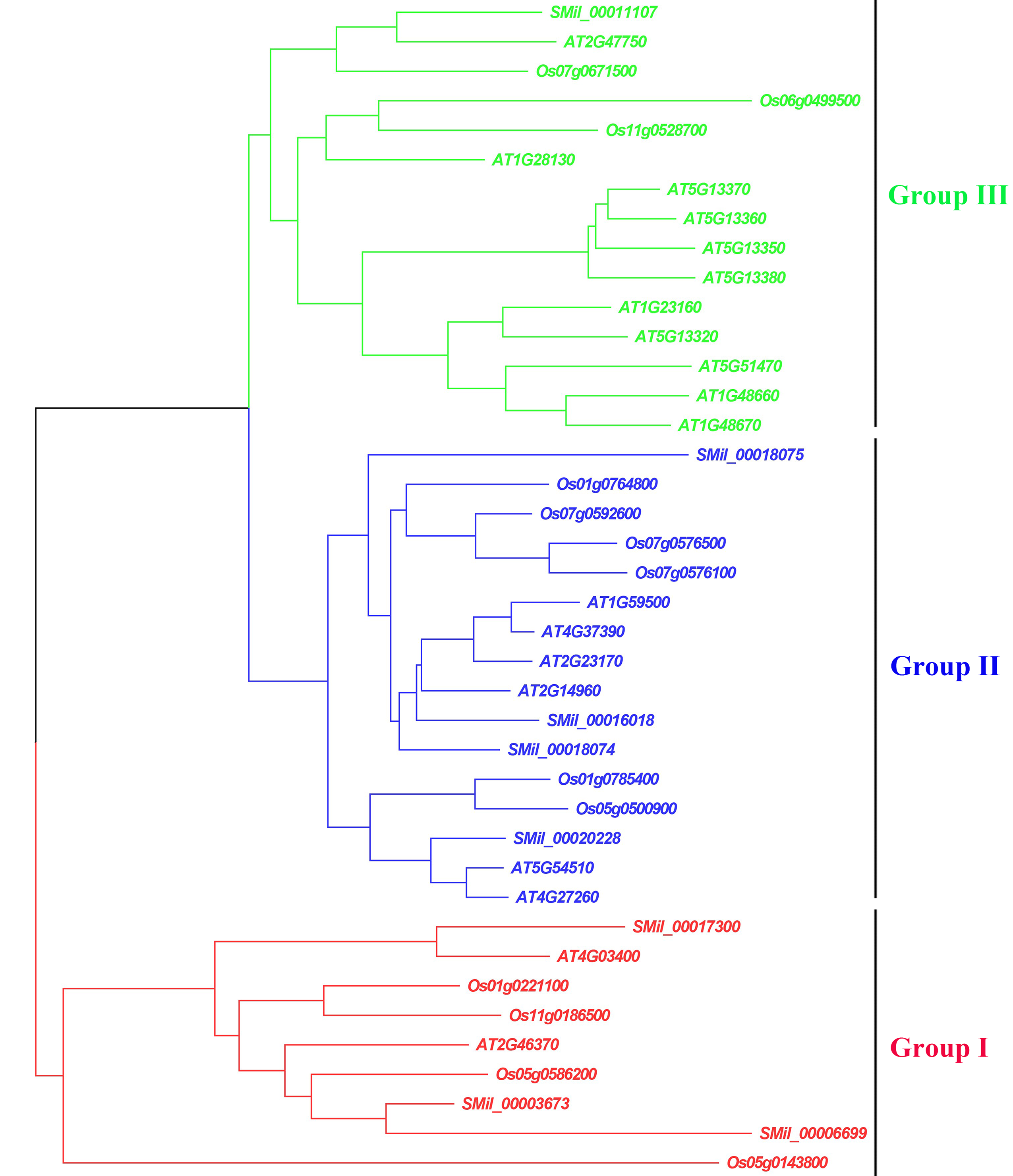

Supplement: Supplementary Figure 2 — The neighbor-joining phylogenetic tree was reconstructed with GH3 amino acid sequences of A. thaliana, S. miltiorrhiza, and O. sativa using MEGA 6.0. [file Image2.tif]

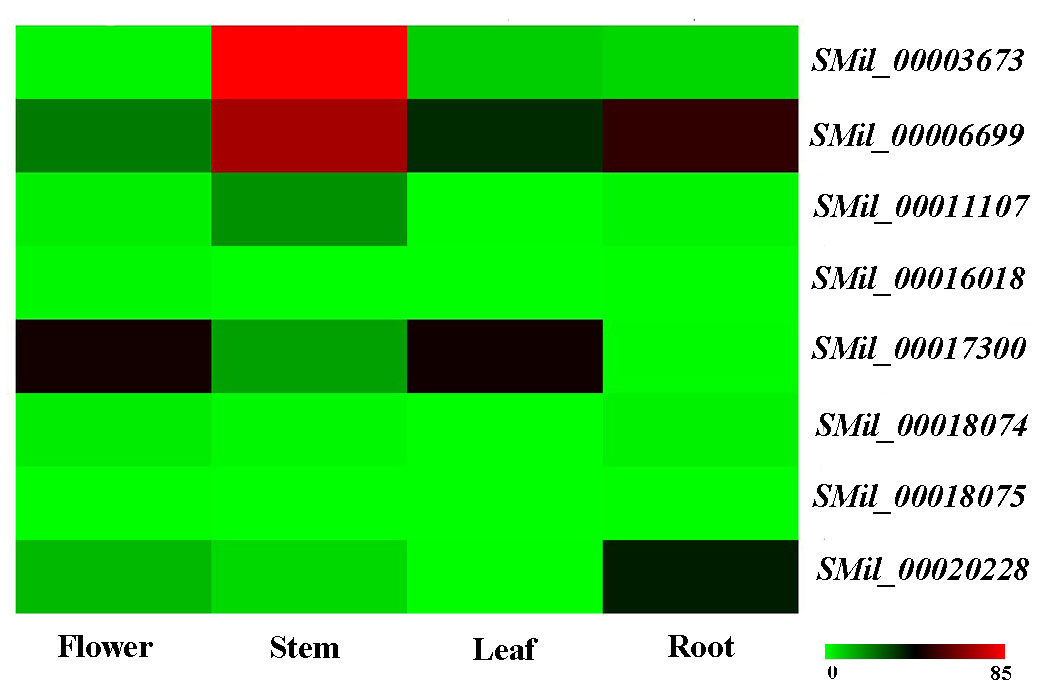

Supplement: Supplementary Figure 3 — Heatmaps representing the expression profiles of SmGH3, genes in the Flower, Leaf, Root and Stem. [file Image3.tif]

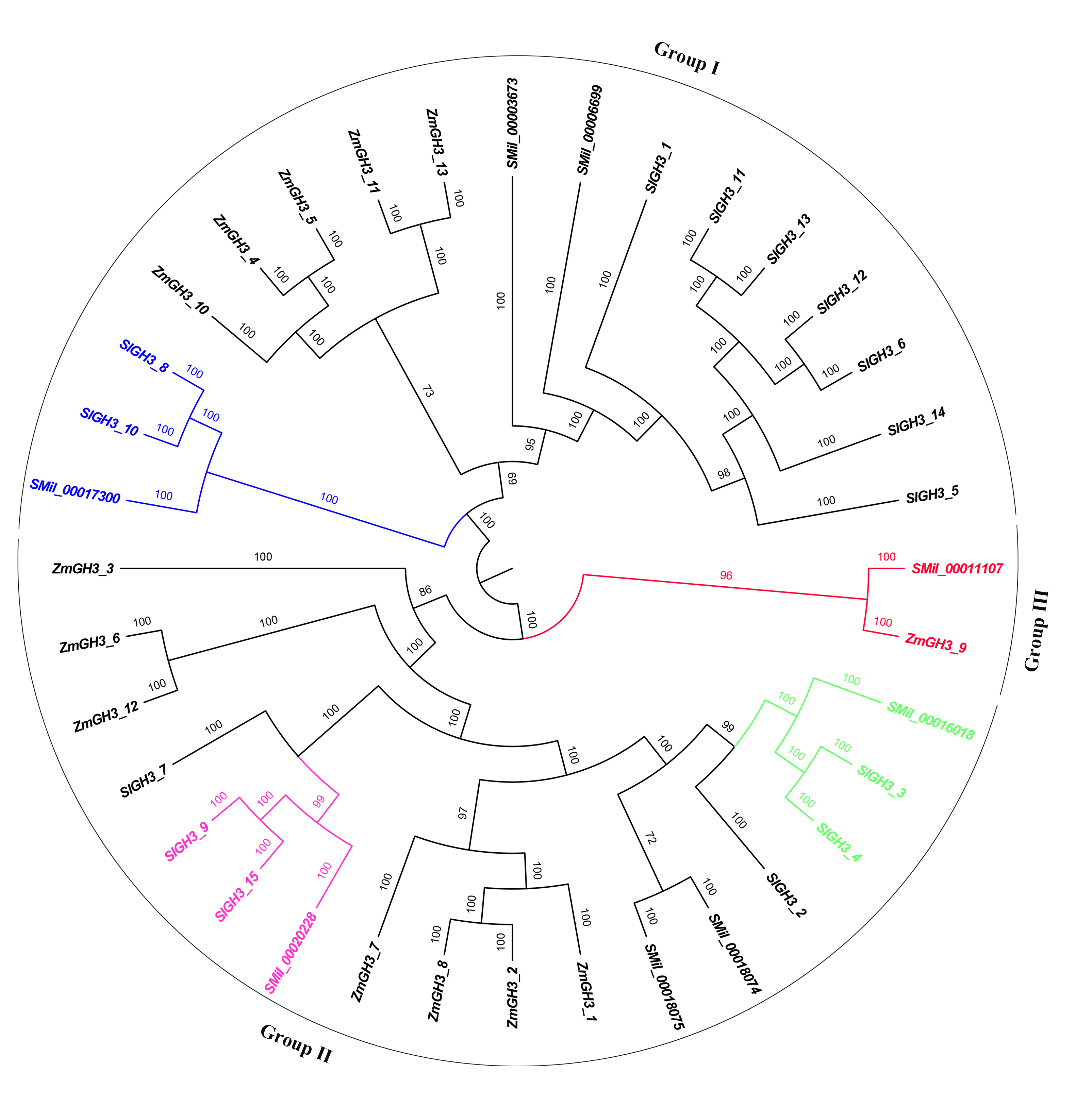

Supplement: Supplementary Figure 4 — The phylogenetic tree for the GH3 gene family in maize (Zea mays), tomato (Solanum lycopersicum), and S. miltiorrhiza. [file Image4.tif]
