# Supplementary material for: Genome-wide comprehensive analysis the molecular phylogenetic evolution, functional divergence and tissue-specific expression of GH3 gene family in Salvia miltiorrhiza, Arabidopsis thaliana, and Oryza sativa
Source: Front Plant Sci. 2025 Nov 14;16:1644853. doi: 10.3389/fpls.2025.1644853 (PMC12661205; doi:10.3389/fpls.2025.1644853)
Supplement: Supplementary file 5 [file Table1.docx]

**Supplementary table 1: Gene features of *GH3* in *S. miltiorrhiza*，*A. thaliana*， and *O. sativa***

| **Gene ID** | **Gene length(bp)** | **CDS length(bp)** | **Protein(aa)** | **Mw(Da)** | **p*I*** |
| --- | --- | --- | --- | --- | --- |
| *SMil_00003673* | 3202 | 1713 | 570 | 62995.82 | 5.92 |
| *SMil_00006699* | 4103 | 1743 | 580 | 64018.60 | 7.20 |
| *SMil_00011107* | 3731 | 1818 | 605 | 67885.58 | 5.59 |
| *SMil_00016018* | 2542 | 1791 | 596 | 66679.80 | 5.57 |
| *SMil_00017300* | 3483 | 1221 | 406 | 45822.81 | 6.11 |
| *SMil_00018074* | 2263 | 1731 | 576 | 65130.79 | 6.37 |
| *SMil_00018075* | 1996 | 1839 | 612 | 68679.85 | 6.22 |
| *SMil_00020228* | 2285 | 1785 | 594 | 66862.24 | 5.93 |
| *AT1G59500* | 2122 | 1794 | 597 | 67046.28 | 5.38 |
| *AT2G46370* | 2705 | 1761 | 586 | 65677.10 | 5.78 |
| *AT2G47750* | 3834 | 1758 | 585 | 66158.49 | 6.08 |
| *AT5G54510* | 2725 | 1839 | 612 | 68896.52 | 5.53 |
| *AT5G13380* | 2603 | 1872 | 624 | 70274.57 | 5.53 |
| *AT5G13370* | 2924 | 1788 | 595 | 66978.68 | 5.49 |
| *AT5G51470* | 2339 | 1746 | 581 | 65765.98 | 5.18 |
| *AT4G03400* | 4064 | 1776 | 591 | 66859.17 | 5.74 |
| *AT4G37390* | 2405 | 1812 | 603 | 68159.93 | 5.89 |
| *AT2G23170* | 2708 | 1788 | 595 | 67536.599 | 5.98 |
| *AT4G27260* | 2707 | 1839 | 612 | 69283.34 | 5.72 |
| *AT2G14960* | 2479 | 1773 | 590 | 66734.33 | 5.98 |
| *AT1G23160* | 2261 | 1737 | 578 | 65617.98 | 5.32 |
| *AT1G48660* | 2025 | 1722 | 573 | 64124.93 | 5.01 |
| *AT5G13360* | 2987 | 2019 | 672 | 75873.03 | 6.08 |
| *AT1G28130* | 2950 | 1830 | 609 | 68864.03 | 5.11 |
| *AT5G13320* | 3609 | 1728 | 575 | 65128.13 | 4.91 |
| *AT1G48670* | 2374 | 1578 | 525 | 59142.59 | 5.17 |
| *AT5G13350* | 2627 | 1764 | 587 | 66375.21 | 5.52 |
| *Os01g0785400* | 2944 | 1833 | 610 | 67366.96 | 6.45 |
| *Os01g0764800* | 3748 | 1845 | 614 | 67975.90 | 5.73 |
| *Os01g0221100* | 5501 | 1776 | 591 | 64190.96 | 5.90 |
| *Os05g0500900* | 2571 | 1890 | 629 | 68788.62 | 5.83 |
| *Os05g0586200* | 5520 | 1746 | 581 | 65095.10 | 5.42 |
| *Os05g0143800* | 2312 | 1809 | 602 | 65243.61 | 6.87 |
| *Os06g0499500* | 3784 | 1863 | 620 | 69020.30 | 5.32 |
| *Os07g0592600* | 2431 | 1818 | 605 | 66931.57 | 5.60 |
| *Os07g0576500* | 2031 | 1326 | 441 | 48463.24 | 5.22 |
| *Os07g0576100* | 4673 | 1437 | 478 | 52279.65 | 6.06 / |
| *Os07g0671500* | 8610 | 1722 | 573 | 64723.51 | 5.63 |
| *Os11g0186500* | 2031 | 1842 | 613 | 66659.42 | 5.79 |
| *Os11g0528700* | 1561 | 1410 | 469 | 50964.09 | 5.03 |
