# Supplementary material for: Genome-wide comprehensive analysis the molecular phylogenetic evolution, functional divergence and tissue-specific expression of GH3 gene family in Salvia miltiorrhiza, Arabidopsis thaliana, and Oryza sativa
Source: Front Plant Sci. 2025 Nov 14;16:1644853. doi: 10.3389/fpls.2025.1644853 (PMC12661205; doi:10.3389/fpls.2025.1644853)
Supplement: Supplementary file 7 [file Table3.docx]

**Supplementary Table 3: Normal expression sequences of 22 motifs identified in 40 GH3 proteins in *A. thaliana*, *S. miltiorrhiza* and O. sativa**

| **Motif** | **Length(aa)** | **Frequency** | **E-value** | **Normal expression sequences** |
| --- | --- | --- | --- | --- |
| 1 | 50 | 37 | 4.2e-1238 | YELV[VI]TT[YF][AS]GLYRYR[VL]GD[VI][LV]RV[TA]GF[HY]N[NAS][AT]PQF[RK]F[VI][RC]R[KR]NV[VL]LSIDSDKTD |
| 2 | 41 | 40 | 8.7e-1012 | LPL[VAI][SC]TMY[AG]SSE[CS]Y[FI]G[IVL]N[LV][NR]P[LM]C[KP]P[SE][ED]VS[YF]T[IL][LIM]PNM[AG]YFEF |
| 3 | 50 | 39 | 6.9e-1106 | PD[EA][AV]ILCPDSXQS[ML]Y[CA][QH][LM]LCGL[LV]QRDEVLR[VL]GAVFAS[GAS][LF][LV]RAI[RK]FL[EQ]L[HN]W |
| 4 | 41 | 40 | 2.4e-1004 | CSKX[SN]WEGII[TP]R[LI]WP[NK][AT]KY[IVL][DE][VA][IV][VM]TG[SAT]MAQYIP[TK]L[ER][FYH]Y[SA][GN]G |
| 5 | 29 | 37 | 1.4e-555 | [KA]D[KG]SIGPLEI[RK]VV[RK]PG[TA]F[DE][EK]LMD[YF][AF][IVL]S[RL]G |
| 6 | 29 | 39 | 7.9e-560 | FK[KS][KN]VPVV[TS]Y[ED]D[LIV]KPYIQR[IV]A[ND]G[DE][RP]S[PD][ILV][LI] |
| 7 | 41 | 27 | 8.6e-551 | [DE]RR[QT][LF]LYSLLMP[VI]M[NS][KL]Y[VI]PGLD[KE]GK[GA][ML]Y[FL][LY]F[VI]KSE[ST]KTP[GS]G |
| 8 | 34 | 38 | 2.7e-513 | [AEK]ALE[FL][ILF]E[ED][LVM]T[RT]N[AV]DQ[VI]Q[RDE]E[VT]L[ER]EIL[SAT]RN[AS]G[TA]EYL |
| 9 | 29 | 39 | 8.4e-511 | [STC][AGS][HK]PI[TS]E[FL][LS][TL]SSGT[ST][AG]G[EK][RP]KLMP[TF][INT][ED]E[ED]L |
| 10 | 35 | 21 | 3.5e-349 | [AS]SINQYK[TV]PRCV[TKS]FT[PE][IA][VL]E[LI]L[DE]SRVVSS[HF]FSP[KAS][LC]P |
| 11 | 15 | 34 | 1.7e-303 | ADTS[ST]IPGHYV[LIV][YF]WE |
| 12 | 21 | 22 | 1.0e-230 | SYYKSD[HY]FKNRPYDPY[NT][VN]YTS |
| 13 | 25 | 37 | 2.0e-282 | IRTGTLSS[WR][IV]TDP[SA][CVI]REA[VM][SG]KILR[PA] |
| 14 | 15 | 29 | 3.2e-192 | [ER]CCL[AE][VM]EESL[ND][SA]VYR |
| 15 | 21 | 34 | 3.1e-167 | VE[NA]A[SK]LLL[ER][PE]X[GD][AL][MS][LV][VM][ED][YF]TSY |
| 16 | 15 | 39 | 9.2e-158 | EXE[LVP]V[DE]L[VA]DV[KE][VLI]GK[EY] |
| 17 | 39 | 8 | 3.1e-097 | [PI][IRV][EDG]NG[KR][AV]L[QE]FIY[SG]S[KR][QE][FY][KI]TKGG[LI]T[AV][GT]TATT[NH][VL]Y[RA][SN][EA]EFK |
| 18 | 11 | 36 | 1.0e-084 | [ND]PELADF[IV]EQE |
| 19 | 21 | 16 | 1.3e-084 | [AGS]S[VI][NSG]Q[YF]KTPRC[VI]K[SP]SNALV[LV][LQ] |
| 20 | 11 | 25 | 3.0e-045 | RF[FG]L[ND]G[AR]TD[RK][EK] |
| 21 | 8 | 19 | 5.3e-039 | [LPARPVLT |
| 22 | 15 | 7 | 2.2e-027 | [TNS]T[LM]QI[YF][RK][TL][SA][FAY]A[FY]R[NS]R |
