# Supplementary material for: Genome-wide comprehensive analysis the molecular phylogenetic evolution, functional divergence and tissue-specific expression of GH3 gene family in Salvia miltiorrhiza, Arabidopsis thaliana, and Oryza sativa
Source: Front Plant Sci. 2025 Nov 14;16:1644853. doi: 10.3389/fpls.2025.1644853 (PMC12661205; doi:10.3389/fpls.2025.1644853)
Supplement: Supplementary file 8 [file Table4.docx]

**Supplementary Table 4 :The GH3 protein motifs diagram of *A. thaliana, S. miltiorrhiza* and *O. sativa***

| Group | Genes | Motif diagram |
| --- | --- | --- |
| Group I | *SMil_00011107* | 11- [8]-16-[6]-[9]-[7]-[21]-[12]-[3]-6-[13]-1-[18]-1-[4]-1-[2]-24-[16]-[1]-8-[15]-[11]-18-[14]-4-[5]-1-[10]-4 |
|  | *AT2G47750* | 9-[8]-12-[6]-[9]-[7]-[21]-[12]-[3]-6-[13]-1-[18]-[4]-1-[2]-20-[16]-[1]-6-[15]-[11]-11-[14]-4-[5]-[19]-19 |
|  | *Os07g0671500* | 39-[6]-[9]-[7]-[21]-[12]-[3]-6-[13]-1-[18]-[4]-1-[2]-17-[16]-[1]-7-[15]-[11]-18-[14]-4-[5]-[10]-4 |
|  | *AT5G13370* | 11-[8]-[20]-1-[6]-[9]-[7]-28-[3]-6-[13]-1-[18]-[4]-[2]-16-[16]-[1]-7-[15]-[11]-18-[14]-4-[5]-[10]-11 |
|  | *AT5G13360* | 89-[8]-[20]-1-[6]-[9]-[7]-28-[3]-6-[13]-1-[18]-[4]-[2]-15-[16]-[1]-7-[15]-[11]-18-[14]-4-[5]-[10]-11 |
|  | *AT5G51470* | 10-[8]-[20]-1-[6]-[9]-[7]-8-[12]-[3]-6-[13]-1-[18]-[4]-[2]-7-[16]-[1]-7-[15]-[11]-18-[14]-4-[5]-[10]-6 |
|  | *AT1G48660* | 4-[8]-[20]-1-[6]-[9]-48-[12]-[3]-6-[13]-1-[18]-[4]-[2]-8-[16]-[1]-7-[15]-[11]-18-[14]-4-[5]-[19]-18 |
|  | *AT1G48670* | 5-[8]-[20]-1-[6]-[9]-20-[3]-6-[13]-1-[18]-[4]-[2]-8-[16]-[1]-7-[15]-[11]-18-[14]-4-[5]-[10]-4 |
|  | *AT1G23160* | 12-[8]-12-[6]-[9]-[7]-8-[12]-[3]-6-[13]-1-[18]-[4]-1-[2]-6-[16]-[1]-7-[15]-[11]-22-[14]-4-[5]-[19]-11 |
|  | *AT5G13320* | 9-[8]-12-[6]-[9]-[7]-8-[12]-[3]-6-[13]-1-[18]-[4]-[2]-7-[16]-[1]-7-[15]-[11]-22-[14]-4-[5]-[19]-11 |
|  | *AT1G28130* | 11-[8]-[20]-3-[6]-[9]-[7]-[21]-[12]-[3]-6-[13]-1-[18]-[4]-[2]-27-[16]-[1]-9-[15]-[11]-18-[14]-4-[5]-[10]-9 |
|  | *Os06g0499500* | 14-[8]-[20]-6-[6]-2-[9]-7-[7]-[21]-[12]-[3]-6-[13]-2-[18]-[4]-[2]-22-[16]-[1]-8-[15]-[11]-19-[14]-3-[5]-[10]-10 |
|  | *Os11g0528700* | 44-[8]-21-[6]-[9]-[7]-[21]-[12]-[3]-7-[13]-2-[18]-[4]-[2]-13-[16]-37 |
| Group II | *SMil_00016018* | 16-[8]-1-[20]-1-[6]-[9]-[7]-[21]-[12]-[3]-6-[13]-[18]-[4]-[2]-18-[16]-[1]-7-[15]-[11]-14-[14]-4-[5]-[10]-8 |
|  | *AT1G59500* | 21-[8]-1-[20]-1-[6]-[9]-[7]-[21]-19-[3]-6-[13]-1-[18]-[4]-[2]-12-[16]-[1]-7-[15]-[11]-17-[14]-4-[5]-[10]-8 |
|  | *AT4G37390* | 21-[8]-1-[20]-1-[6]-[9]-[7]-[21]-[12]-[3]-6-[13]-1-[18]-[4]-[2]-16-[16]-[1]-7-[15]-[11]-17-[14]-4-[5]-[10]-8 |
|  | *AT2G23170* | 20-[8]-1-[20]-1-[6]-[9]-[7]-[21]-[12]-[3]-6-[13]-1-[18]-1-[4]-[2]-9-[16]-[1]-7-[15]-[11]-16-[14]-4-[5]-[10]-8 |
|  | *AT2G14960* | 20-[8]-1-[20]-1-[6]-[9]-[7]-[21]-[12]-[3]-6-[13]-[18]-[4]-[2]-4-[16]-[1]-8-[15]-[11]-16-[14]-4-[5]-[10]-9 |
|  | *SMil_00018074* | 10-[8]-1-[20]-1-[6]-[9]-[7]-[21]-[12]-[3]-6-[13]-[18]-[4]-[2]-5-[16]-[1]-7-[15]-[11]-13-[14]-4-[5]-[10]-8 |
|  | *SMil_00018075* | 44-[8]-1-[20]-1-[6]-[9]-[7]-8-[12]-[3]-6-[13]-[18]-[4]-[2]-5-[16]-[1]-7-[15]-27-[14]-6-[5]-[10]-9 |
|  | *Os01g0764800* | 30-[8]-1-[20]-1-[6]-[9]-[7]-[21]-[12]-[3]-6-[13]-1-[18]-[4]-[2]-15-[16]-[1]-7-[15]-[11]-22-[14]-3-[5]-[10]-7 |
|  | *Os07g0592600* | 27-[8]-1-[20]-1-[6]-[9]-[7]-[21]-[12]-[3]-6-[13]-[18]-[4]-[2]-12-[16]-[1]-7-[15]-[11]-17-[14]-4-[5]-[10]-9 |
|  | *Os07g0576500* | 12-[21]-17-[3]-6-[13]-[18]-[4]-[2]-8-[16]-[1]-8-[15]-[11]-21-[14]-4-[5]-[10]-9 |
|  | *Os07g0576100* | 29-[8]-14-[6]-[9]-[7]-[21]-16-[3]-23-[4]-[2]-8-[16]-[1]-8-[15]-21 |
|  | *SMil_00020228* | 21-[8]-1-[20]-1-[6]-[9]-[7]-[21]-[12]-[3]-6-[13]-[18]-[4]-[2]-10-[16]-[1]-7-[15]-[11]-15-[14]-3-[5]-[10]-9 |
|  | *AT5G54510* | 22-[8]-1-[20]-1-[6]-[9]-[7]-[21]-[12]-[3]-6-[13]-[18]-[4]-[2]-23-[16]-[1]-7-[15]-[11]-15-[14]-4-[5]-[10]-12 |
|  | *AT4G27260* | 22-[8]-1-[20]-1-[6]-[9]-[7]-[21]-[12]-[3]-6-[13]-[18]-[4]-[2]-23-[16]-[1]-7-[15]-[11]-15-[14]-4-[5]-[10]-12 |
|  | *Os01g0785400* | 21-[8]-14-[6]-[9]-[7]-[21]-[12]-[3]-6-[13]-[18]-[4]-[2]-13-[16]-[1]-7-[15]-[11]-14-[14]-4-[5]-[10]-21 |
|  | *Os05g0500900* | 24-[8]-15-[6]-[9]-[7]-[21]-[12]-[3]-6-[13]-1-[18]-[4]-[2]-16-[16]-[1]-7-[15]-1-[11]-16-[14]-4-[5]-[10]-29 |
| Group III | *Os05g0143800* | 8-[8]-25-[6]-1-[9]-22-[17]-11-[3]-34-[18]-[4]-1-[2]-13-[16]-[1]-63-[14]-3-[5]-[19]-17 |
|  | *SMil_00017300* | 7-[8]-20-[6]-[9]-1-[22]-3-[17]-12-[3]-6-[13]-[18]-3-[4]-[2]-15-[16]-10 |
|  | *AT4G03400* | 9-[8]-20-[6]-[9]-1-[22]-3-[17]-12-[3]-6-[13]-[18]-4-[4]-[2]-19-[16]-[1]-8-[15]-[11]-30-[5]-[19]-14 |
|  | *Os01g0221100* | 18-[8]-1-[20]-1-[6]-[9]-1-[22]-2-[17]-12-[3]-7-[13]-1-[18]-1-[4]-[2]-13-[16]-[1]-8-[15]-[11]-34-[5]-[19]-16 |
|  | *Os11g0186500* | 15-[8]-13-[6]-[9]-1-[22]-2-[17]-15-[3]-7-[13]-1-[18]-1-[4]-[2]-29-[16]-[1]-33-[11]-14-[14]-5-[5]-[19]-18 |
|  | *AT2G46370* | 22-[8]-15-[6]-[9]-1-[22]-3-[17]-12-[3]-6-[13]-[18]-1-[4]-[2]-9-[16]-[1]-7-[15]-[11]-30-[5]-[19]-15 |
|  | *Os05g0586200* | 7-[8]-1-[20]-1-[6]-[9]-1-[22]-2-[17]-12-[3]-6-[13]-[18]-1-[4]-[2]-20-[16]-[1]-7-[15]-[11]-30-[5]-[19]-17 |
|  | *SMil_00003673* | 12-[8]-1-[20]-1-[6]-2-[9]-1-[22]-2-[17]-12-[3]-6-[13]-[18]-1-[4]-[2]-4-[16]-[1]-7-[15]-[11]-30-[5]-[19]-15 |
|  | *SMil_00006699* | 12-[8]-1-[20]-1-[6]-[9]-1-[22]-2-[17]-12-[3]-6-[13]-[18]-1-[4]-[2]-12-[16]-127-[5]-[19]-15 |
