# Supplementary material for: Genome-wide comprehensive analysis the molecular phylogenetic evolution, functional divergence and tissue-specific expression of GH3 gene family in Salvia miltiorrhiza, Arabidopsis thaliana, and Oryza sativa
Source: Front Plant Sci. 2025 Nov 14;16:1644853. doi: 10.3389/fpls.2025.1644853 (PMC12661205; doi:10.3389/fpls.2025.1644853)
Supplement: Supplementary file 10 [file Table6.docx]

**Supplementary Table 6: Ka/Ks and divergence analysis of *GH3* paralogous in *A. thaliana, S. miltiorrhiza,* and *O. sativa***

| **Genes** | ***Ka*** | ***Ks*** | ***Ka*/*Ks*** | **Purifying selection** | **Subgroup** | **Protein identity (%)** |
| --- | --- | --- | --- | --- | --- | --- |
| *AT1G48670*  *AT1G48660* | 0.1544 | 0.4747 | 0.3252 | yes | Group Ⅲ | 68.06 |
| *AT1G23160*  *AT5G13320* | 0.1596 | 0.7659 | 0.2084 | yes | Group Ⅲ | 73.92 |
| *AT5G13350*  *AT5G13380* | 0.1369 | 0.3292 | 0.4158 | yes | Group Ⅲ | 71.36 |
| *AT1G59500*  *AT4G37390* | 0.0579 | 0.2468 | 0.2346 | yes | GroupⅡ | 87.73 |
| *AT5G54510*  *AT4G27260* | 0.0562 | 0.7818 | 0.0718 | yes | GroupⅡ | 90.36 |
| [*Os06g0499500*](https://rapdb.dna.affrc.go.jp/viewer/gbrowse_details/irgsp1?name=Os06g0499500) *[Os11g0528700](https://rapdb.dna.affrc.go.jp/viewer/gbrowse_details/irgsp1?name=Os11g0528700)* | 0.3511 | 7.5369 | 0.0466 | yes | Group Ⅲ | 35.06 |
| [*Os07g0576500*](https://rapdb.dna.affrc.go.jp/viewer/gbrowse_details/irgsp1?name=Os07g0576500) *[Os07g0576100](https://rapdb.dna.affrc.go.jp/viewer/gbrowse_details/irgsp1?name=Os07g0576100)* | 0.0833 | 0.6222 | 0.1338 | yes | GroupⅡ | 41.82 |
| [*Os01g0785400*](https://rapdb.dna.affrc.go.jp/viewer/gbrowse_details/irgsp1?name=Os01g0785400) *[Os05g0500900](https://rapdb.dna.affrc.go.jp/viewer/gbrowse_details/irgsp1?name=Os05g0500900)* | 0.0979 | 2.0114 | 0.0487 | yes | GroupⅡ | 78.86 |
| [*Os01g0221100*](https://rapdb.dna.affrc.go.jp/viewer/gbrowse_details/irgsp1?name=Os01g0221100) *[Os11g0186500](https://rapdb.dna.affrc.go.jp/viewer/gbrowse_details/irgsp1?name=Os11g0186500)* | 0.1765 | 1.6488 | 0.1071 | yes | GroupⅠ | 64.34 |
| *SMil_00018074*  *SMil_00018075* | 0.2687 | 1.1699 | 0.2297 | yes | Group Ⅱ | 56.17 |
| *SMil_00003673*  *SMil_00006699* | 0.2835 | 3.5370 | 0.0802 | yes | Group Ⅰ | 58.83 |
