# Supplementary material for: Genome-wide comprehensive analysis the molecular phylogenetic evolution, functional divergence and tissue-specific expression of GH3 gene family in Salvia miltiorrhiza, Arabidopsis thaliana, and Oryza sativa
Source: Front Plant Sci. 2025 Nov 14;16:1644853. doi: 10.3389/fpls.2025.1644853 (PMC12661205; doi:10.3389/fpls.2025.1644853)
Supplement: Supplementary file 11 [file Table7.docx]

**Supplementary Table 7: The coefficient of Type-I functional divergence (*θ*_I_) from pairwise comparisons between *GH3* groups of *A. thaliana, S. miltiorrhiza* and *O. sativa***

| **Category** | **Coefficient of typeI functional divergence (θ_I_)±standard error** | **LRT(likelihood ratio statistic )** | ***P*-value** | **Positive selection sites**  **(0.8 > Qk > 0.67)** | **Positive selection sites**  **(Qk > 0.8)** |
| --- | --- | --- | --- | --- | --- |
| **GroupI vs. Group II** | 0.561910 ± 0.101682 | 58.385064 | < 0.01^**^ | 318、418、464、400  354、325、427、360 | 436、518、443、333、423、452、337、469、428、433、254、355、324、361、343、391、358、322、404 |
| **Group I vs. Group** **III** | 0.440390 ± 0.101014 | 30.421918 | < 0.01^**^ | 476、400、362、401  350、430、365 | 324、317、443、424、425、391、361、322、455 |
| **Group II vs. Group III** | 0.237761 ± 0.076930 | 8.325800 | < 0.01^**^ | 350、344 | 358 |
