# Supplementary material for: Genome-wide comprehensive analysis the molecular phylogenetic evolution, functional divergence and tissue-specific expression of GH3 gene family in Salvia miltiorrhiza, Arabidopsis thaliana, and Oryza sativa
Source: Front Plant Sci. 2025 Nov 14;16:1644853. doi: 10.3389/fpls.2025.1644853 (PMC12661205; doi:10.3389/fpls.2025.1644853)
Supplement: Supplementary file 12 [file Table8.docx]

**Supplementary Table 8: The coefficient of Type-II functional divergence (*θ*_II_) from pairwise comparisons between GH3 groups of *A. thaliana, S. miltiorrhiza* and *O. sativa***

| **Category** | **Coefficient of typeIIfunctional divergence**  **(*θ*_II_)±standard error** | ***P*-value** | **Positive selection sites**  **(Qk > 0.9)** | **Positive selection sites**  **(Qk > 1)** |
| --- | --- | --- | --- | --- |
| **Group I vs. Group II** | -0.136874 ± 0.205093 | 0.25140 | 0 | 0 |
| **Group I vs. Group III** | -0.637158 ± 0.317292 | 0.02275^*^ | 0 | 0 |
| **Group II vs. Group III** | -0.283415 ± 0.208297 | 0.08691 | 0 | 0 |
