# Supplementary material for: Genome-wide comprehensive analysis the molecular phylogenetic evolution, functional divergence and tissue-specific expression of GH3 gene family in Salvia miltiorrhiza, Arabidopsis thaliana, and Oryza sativa
Source: Front Plant Sci. 2025 Nov 14;16:1644853. doi: 10.3389/fpls.2025.1644853 (PMC12661205; doi:10.3389/fpls.2025.1644853)
Supplement: Supplementary file 13 [file Table9.docx]

**Supplementary Table 9: The distribution for the ESTs of *GH3* genes in *A. thaliana***

| **Genes** | **Number of ESTs** | **Tissue** | | | | | |
| --- | --- | --- | --- | --- | --- | --- | --- |
|  |  | **Rosette** | **Seedling** | **Root** | **Inflorescence** | **Callus** | **Cell Suspension** |
| *AT1G59500* | 0 |  |  |  |  |  |  |
| *AT2G46370* | 52 |  |  | **+** | **+** |  | **+** |
| *AT2G47750* | 11 |  | **+** |  | **+** |  |  |
| *AT5G54510* | 28 |  |  | **+** | **+** |  |  |
| *AT5G13380* | 0 |  |  |  |  |  |  |
| *AT5G13370* | 6 |  | **+** |  |  |  |  |
| *AT5G51470* | 0 |  |  |  |  |  |  |
| *AT4G03400* | 14 | **+** | **+** | **+** | **+** |  |  |
| *AT4G37390* | 24 |  | **+** | **+** |  |  |  |
| *AT2G23170* | 26 | **+** |  | **+** | **+** |  |  |
| *AT4G27260* | 67 | **+** |  | **+** | **+** |  |  |
| *AT2G14960* | 1 |  |  | **+** |  |  |  |
| *AT1G23160* | 0 |  |  |  |  |  |  |
| *AT1G48660* | 1 |  |  |  |  |  |  |
| *AT5G13360* | 5 | **+** |  | **+** |  |  |  |
| *AT1G28130* | 20 | **+** |  | **+** | **+** |  |  |
| *AT5G13320* | 13 | **+** |  | **+** | **+** |  |  |
| *AT1G48670* | 0 |  |  |  |  |  |  |
| *AT5G13350* | 1 |  | **+** |  |  |  |  |
