# Supplementary material for: Genome-wide comprehensive analysis the molecular phylogenetic evolution, functional divergence and tissue-specific expression of GH3 gene family in Salvia miltiorrhiza, Arabidopsis thaliana, and Oryza sativa
Source: Front Plant Sci. 2025 Nov 14;16:1644853. doi: 10.3389/fpls.2025.1644853 (PMC12661205; doi:10.3389/fpls.2025.1644853)
Supplement: Supplementary file 14 [file Table10.docx]

**Supplementary Table 10: The distribution for the ESTs of *GH3* genes in** ***O. sativa***

| **Genes** | **Number of ESTs** | **Tissue** | | | | | |
| --- | --- | --- | --- | --- | --- | --- | --- |
|  |  | **Rosette** | **Seedling** | **Root** | **Inflorescence** | **Callus** | **Cell Suspension** |
| *Os01g0785400* | 15 |  | **+** |  | **+** | **+** |  |
| *Os01g0764800* | 96 |  |  | **+** | **+** | **+** |  |
| *Os01g0221100* | 19 |  |  |  | **+** | **+** |  |
| *Os05g0500900* | 99 |  | **+** |  |  | **+** |  |
| *Os05g0586200* | 43 |  |  |  | **+** | **+** |  |
| *Os05g0143800* | 26 |  | **+** |  |  | **+** |  |
| *Os06g0499500* | 28 |  |  | **+** | **+** | **+** |  |
| *Os07g0592600* | 96 |  | **+** |  | **+** | **+** |  |
| *Os07g0576500* | 1 |  |  |  |  |  |  |
| *Os07g0576100* | 0 |  |  |  |  |  |  |
| *Os07g0671500* | 23 |  | **+** |  | **+** | **+** |  |
| *Os11g0186500* | 0 |  |  |  |  |  |  |
| *Os11g0528700* | 0 |  |  |  |  |  |  |
