# Supplementary material for: Genome-wide comprehensive analysis the molecular phylogenetic evolution, functional divergence and tissue-specific expression of GH3 gene family in Salvia miltiorrhiza, Arabidopsis thaliana, and Oryza sativa
Source: Front Plant Sci. 2025 Nov 14;16:1644853. doi: 10.3389/fpls.2025.1644853 (PMC12661205; doi:10.3389/fpls.2025.1644853)
Supplement: Supplementary file 15 [file Table11.docx]

**Supplementary Table 11:** The RPKM values for *S. miltiorrhiza* organs of Flower, Stem, Leaf and Root

| **Gene ID** | **Flower** | **Stem** | **Leaf** | **Root** |
| --- | --- | --- | --- | --- |
| *SMil_00003673* | 1.58109 | 84.2331 | 7.99633 | 6.68776 |
| *SMil_00006699* | 21.8362 | 69.4762 | 34.8158 | 50.0709 |
| *SMil_00011107* | 2.60619 | 18.04 | 0.174049 | 1.48376 |
| *SMil_00016018* | 0.897661 | 0 | 0 | 0.0974441 |
| *SMil_00017300* | 45.3131 | 15.4202 | 44.9027 | 0.615715 |
| *SMil_00018074* | 3.07932 | 1.02234 | 0 | 2.11418 |
| *SMil_00018075* | 0.0188346 | 0 | 0 | 0.0180916 |
| *SMil_00020228* | 11.4759 | 6.23081 | 0.219837 | 37.1326 |
